# The effect of polarity and hydrogen bonding on the electronic and vibrational structure of the salicylate anion in acetonitrile and water: implicit and explicit solvation approaches†

**DOI:** 10.1039/d4ra04606d

**Published:** 2024-09-18

**Authors:** Siddharth Mall Bishen, Meena Adhikari, Sandeep Pokharia, Hirdyesh Mishra

**Affiliations:** a Department of Physics, Institute of Science, Banaras Hindu University Varanasi 221005 India; b Physics Section, MMV, Department of Physics, Banaras Hindu University Varanasi 221005 India hmishra@bhu.ac.in +919454161037; c Chemistry Section, MMV, Banaras Hindu University Varanasi 221005 India

## Abstract

This study investigates the fluorescence quenching of the salicylate anion in water compared to acetonitrile (ACN) and the stability of its *keto* structure in ACN using DFT and TD-DFT methods at the 6-311++G(d,p) basis set. Computational simulations in implicit and explicit environments of ACN and water reveal the effects of solvent polarity and hydrogen bonding on *enol* [O_d_-H⋯O_a_] and *keto* [O_d_⋯H-O_a_] tautomerization, fluorescence quenching, and the spectral profile of the salicylate anion. Implicit solvation models show a barrier height of approximately 1.9 kcal mol^−1^ in ACN and 3.6 kcal mol^−1^ in water for *enol–keto* tautomerization in the ground state, with no barrier in the excited state, leading to an ESIPT reaction in both solvents, but only ground state proton transfer in ACN. Simulated absorption spectra for both *enol* and *keto* forms are similar in both solvents, while the emission spectrum is red-shifted in water. Explicit solvation studies indicate greater stabilization of the salicylate anion in water than in ACN, with a blue shift in absorption and emission spectra and varying oscillator strengths. Solvent molecule positioning affects *enol–keto* stabilization in the ground state, but only the *keto* structure is stabilized in the excited state. Simulated IR spectra in water show a blue shift in O_d_-H stretching frequency and increased water molecule vibrational frequencies, suggesting non-radiative excitation energy transfer from salicylate ions to water molecules *via* n → σ* intermolecular hydrogen bonding interactions. This mechanism explains the fluorescence quenching observed in water and results align with experimental data, indicating hydrogen-bonded *keto* form stabilization both in water and ACN.

## Abstract 2

This study uses computational models to explore how solvent polarity and hydrogen bonding affect sodium salicylate anion in acetonitrile and water to investigate their impact on *enol–keto* stabilization and ESIPT fluorescence quenching.

## Graphical abstract

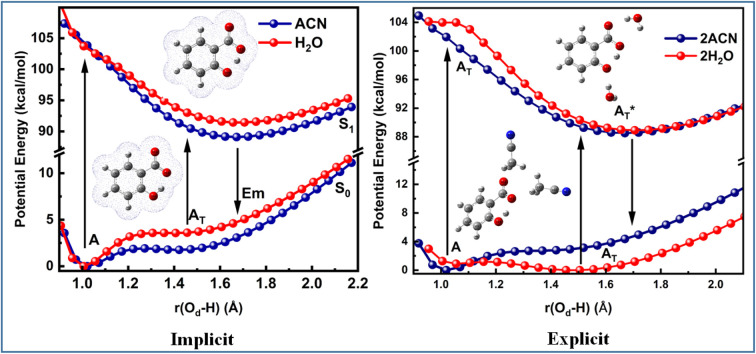

## 1. Introduction

Recent advancements in quantum chemical calculations have made it possible to determine the electronic structures of the ground and excited states, as well as to computationally simulate electronic spectra.^1,2^ These tools are powerful for investigating the fluorescence dynamics of excited state proton transfer (ESPT) in small hydrogen-bonded organic molecules.^1–5^ Salicylic acid (SA) is a notable example of such molecules, containing both acidic (carboxylate) and basic (hydroxyl) group positioned adjacent to each other on the benzene ring, allowing for the formation of both intermolecular and intramolecular hydrogen bonds.^6–8^ SA and its derivatives^9–11^ are of significant interest due to their relevance in biological,^12–14^ pharmaceutical,^15–19^ and cosmetic applications,^20^ as well as in the development of sensors,^21^ smart photonic^22^ and memory devices.^23–26^

SA exists in two rotamers, P (intramolecular hydrogen-bonded) and R (non-intramolecular hydrogen bonded), in non-polar solvents at low concentrations. At higher concentrations, it forms dimers.^27–31^ In polar solvents, SA dissociates to form anions and intermolecular hydrogen-bonded species.^21,32^ Upon photoexcitation, *enol–keto* tautomerization occurs in these species. Friedrich *et al.*^33^ observed ground state intramolecular proton transfer (GSIPT) in acetonitrile (ACN) due to *enol–keto* tautomerization, whereas in water, only the *enol* form exists in the ground state in sodium salicylate. Joshi *et al.*^34^ reported water-induced fluorescence quenching in the salicylate anion in various protic and aprotic polar solvent mixtures, suggesting that quenching is related to changes in solvent polarity/polarizability and hydrogen-bonding acidity. Similar behavior has been observed in gentisic acid molecules in different solvents.^35–37^

The pioneering work of Weller^38,39^ on the asymmetric double-well potential for the excited state intramolecular proton transfer (ESIPT) reaction in Methyl salicylate has led to numerous studies on the electronic structure and ESIPT reaction of SA and its derivatives. Catalán *et al.*^40^ reported the stabilization of the *enol* structure in the ground state and the *keto* structure in the excited state in 2-hydroxybenzoyl compounds using density functional theory (DFT) and configuration interaction singles (CIS) methods. Intramolecular proton transfer in these compounds is strongly dependent on the distances between donor–acceptor oxygen atoms [O_d_-H⋯O_a_] involved in intramolecular hydrogen bonding. *Ab initio* HF and CIS studies have shown that the deprotonated *enol* form of SA has the highest intramolecular hydrogen bond (IMHB) strength compared to the *enol* forms of neutral and cationic species.^41^ DFT calculations have demonstrated a red shift in absorption maxima with increasing water molecules in SA-water complexes, indicating the presence of solvated species in water.^42^

In a recent experimental photophysical study of sodium salicylate in acetonitrile (ACN) and water solvents, it was observed that the fluorescence decay time behavior changed with longer wavelength excitation. Specifically, as the concentration of the solute increased, there was a noticeable decrease in the solute–solvent interactions.^43^ This observation highlighted the significant influence of polarity and hydrogen-bonding capabilities of the solvent on the photophysics of the salicylate anion. Despite these insights, the precise mechanisms behind the fluorescence quenching of the salicylate anion and the instability of its *keto* form in the water remain subjects of active research. Notably, no comprehensive investigation into the impact of solvent polarity and hydrogen bonding on the electronic structural properties of the salicylate anion has been conducted to date. However, a recent study on the solute–solvent interaction of the indole molecule in water and methylcyclohexane, using implicit and explicit models, demonstrated that solvent reorganization significantly contributes to the stabilization of excited state energies.^44^ Therefore, our current study aims to fill this gap by employing quantum chemical DFT calculations to explore the effects of solvent polarity and hydrogen bonding on the stabilization of *enol–keto* tautomers of salicylate anion in both ground and excited states in ACN and water by applying implicit and explicit solvation approaches, respectively. This article seeks to provide a thorough understanding of the photophysics and photochemistry of salicylate anion, elucidating the underlying mechanisms driving fluorescence quenching and tautomer stability in different solvent environments.

## 2. Methodology

The quantum chemical calculations for the salicylate anion were performed using the Gaussian 09 suite of programs.^45^ To account for the dielectric effect of the solvent, the SMD (solvation model based on density) continuum model was employed implicitly in conjunction with the self-consistent reaction field (SCRF) method. This model considers the interaction between the solute's charge density and a continuum representation of the solvent, incorporating solvent-accessible surface area information to estimate surface tension at the solute–solvent interface. The solvents used were ACN and water, with static dielectric constants of 35.69 and 78.36, respectively, at 298 K, and dynamic dielectric constants (square of the solvent's refractive index at 293 K) of 1.81 and 1.78, respectively. To account for the intermolecular hydrogen bonding, the explicit solvation approach was used in which solvent molecules were positioned near the salicylate anion to explicitly model hydrogen bonding interactions.

Four different functionals, namely B3LYP,^46,47^ CAM-B3LYP,^48^ M06-2X,^49^ and PBE0,^50^ were employed at the 6-311++G(d,p)^45^ basis set for molecular geometry optimization and the stability of structures were confirmed through frequency analysis, as none exhibited negative frequencies. The ground and excited-state electronic structures were further explored using DFT and Time-Dependent DFT (TD-DFT) methods, respectively. The strength of IMHB was determined by comparing the energies of optimized closed and open structures, obtained by rotating the phenolic group by 180° (Δ*E*_IMHB_ = *E*_Open_ − *E*_Closed_). The GSIPT potential energy curve was constructed using the “distinguished coordinate formalism” by increasing the phenolic O_d_-H distance in steps of 0.05 Å for 25 steps, optimizing the geometry at each point, and plotting the resulting energy values. The Franck–Condon (FC) potential energy curves for the first three excited states were generated using the TD-DFT/6-311++G(d,p) method. This was achieved by adding the single-point excitation energies to the various points of GSIPT curve along the reaction coordinate (O_d_-H⋯O_a_). Computational simulations were performed to generate electronic absorption and emission spectra in both implicit and explicit solvation models. NBO Version 3.1 ^51,52^ was used to calculate hyperconjugative stabilization energies ((Δ*E*_ij_^(2)^) for selected NBO pairs from the Fock matrix analysis, providing insights into the role of inter- and intramolecular hydrogen bonding on the electronic structure and proton transfer processes in the salicylate anion. Computational IR analysis was performed to check the effect of solvent polarity and hydrogen bonding on O–H stretching vibrations in salicylate anion.

## 3. Results and discussion

Quantum chemical calculations have been employed computationally to obtain absorption/emission spectra, ground/excited state potential energy (PE) curves, molecular electrostatic potential (MEP), Mulliken charges, Natural Bond Orbital (NBO) analysis, and infrared (IR) spectra. These calculations were conducted in both implicit and explicit environments of ACN and water solvents to understand the effects of solvent polarity and hydrogen bonding on the *enol–keto* tautomerization of salicylate anion electronic structures, respectively. The detailed results of these calculations are discussed below.

### 3.1 Geometry optimization and energetics

#### 3.1.1 In implicit solvation approach

**Fig. 1:**
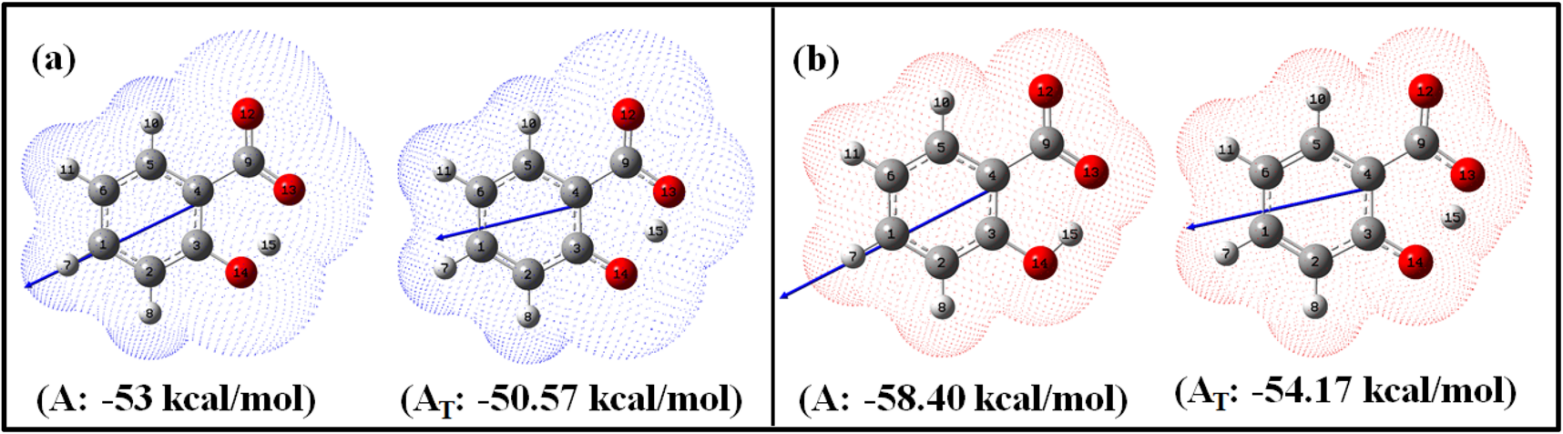
Optimized *enol* (A) and *keto* (A_T_) geometries of salicylate anion in the implicit (a) ACN and (b) water with corresponding solvation energies at B3LYP/6-311++G(d,p) without GD3 correction.

To investigate the influence of the dielectric constants of ACN and water on the molecular properties of the salicylate anion, the *enol* and *keto* forms of the salicylate anion were optimized using the B3LYP/6-311++G(d,p) method, both with and without Grimme's dispersion correction (GD3),^53^ in the ground (S_0_) and excited (S_1_) states within the implicit environment of both solvents. Optimizations were also performed using CAM-B3LYP, M06-2X, and PBE0 in implicit ACN to validate the accuracy and consistency of the results across different functionals. Among all methods tested, B3LYP provided the most energetically stable configuration, as evidenced by its lowest energy value compared to other functionals (ESI Table S1†). Further, the GD3 correction improved stability only by ∼0.0021%. Therefore, B3LYP without GD3 was selected for further calculations due to its balance of accuracy and computational efficiency. The B3LYP/6-311++G(d,p) coordinate data of the optimized structures without GD3 correction in both the solvents in S_0_ and S_1_ states have been tabulated in ESI Table S2–S7.† The optimized structures are shown in Fig. 1 and the geometrically optimized parameters are tabulated in Table 1.

**Table 1:** Optimized parameters of *enol* and *keto* forms of salicylate anion in implicit ACN and water at B3LYP/6-311G++(d,p) without GD3 correction

Solvent	Species	Energy (kcal mol^−1^)	(*μ*) (Debye)	IMHB kcal mol^−1^	*r*(O_d_-H) (Å)	*r*(H-O_a_) (Å)	*r*(O_d_-O_a_) (Å)	∠O_d_-H-O_a_ (°)	Polarizability (a.u.)
ACN	A (*enol*)	5.40	9.887	13.09	1.022	1.525	2.490	155.23°	137.667
	A_T_ (*keto*)	7.16	7.314	15.06	1.415	1.061	2.437	159.47°	142.943
Water	A (*enol*)	0	11.436	7.98	1.006	1.586	2.525	152.90°	142.845
	A_T_ (*keto*)	3.57	8.777	10.65	1.394	1.070	2.425	159.45°	149.223

Fig. 1 illustrates the optimized geometries of *enol* and *keto* forms of salicylate anion structures in both solvents along with its dipole moment vector and corresponding solvation energies. Phenolic oxygen acts as a donor while carboxylic oxygen acts as an acceptor. It is evident from Fig. 1 and Table 1 that the O_d_-H bond length is longer in ACN than in water, accompanied by a contraction in donor to acceptor (O_d_⋯O_a_) distance in *enol* form. In ACN, the proton is detached in its optimized geometry in both *enol* and *keto* forms (Fig. 1a), whereas in water, the proton is bonded to phenolic oxygen in *enol* form while in *keto* form, it is again translocated (Fig. 1b). The solvation energy quantifies the energetic stabilization or destabilization of the molecule when it moves from the gas phase to the solvent phase and is calculated as follows: Δ*E*_sol_ = *E*_(in solvent)_ − *E*_(in gas)_

The negative solvation energies for the *enol* and *keto* forms of the salicylate anion in both solvent environments indicate favorable solvation processes and stabilization of the solute molecules. Water provides greater stabilization to both forms compared to ACN. Further, the difference in solvation energy of the *enol* and *keto* form is higher in water (4.23 kcal mol^−1^) as compared to ACN solvent (2.43 kcal mol^−1^). It suggests that water favors the stabilization of the *enol* form over the *keto* form to a greater extent than ACN. Considering the relative optimized energy in the light of solvation energy (Table 1), it can be concluded that both *enol* and *keto* forms exhibit higher energetic structural stability in water compared to ACN, but in the same solvent, *enol* is a more stable structure than its corresponding *keto* form. Although the *enol* form exhibits a higher dipole moment compared to the *keto* form in both environments, the polarizability of the *keto* form is higher than its corresponding *enol* form. During *enol*–*keto* tautomerization, the change in dipole moment (∼2.6 D) is nearly equal in both solvents.

The IMHB strength of the *enol* form is found greater in ACN than in water. Stronger IMHB would lead to increased acidity of the phenol group or basicity of the carbonyl group resulting in easier proton transfer. With increasing IMHB strength, donor to an acceptor (O_d_⋯O_a_) distance must contract followed by elongation in O_d_-H bond length. This is in accordance with the observed elongation of r(O_d_-H) of salicylate anion (Table 1). Consequently, the likelihood of *enol–keto* conversion is greater in ACN than in water.

#### 3.1.2 In explicit solvation approach

**Fig. 2:**
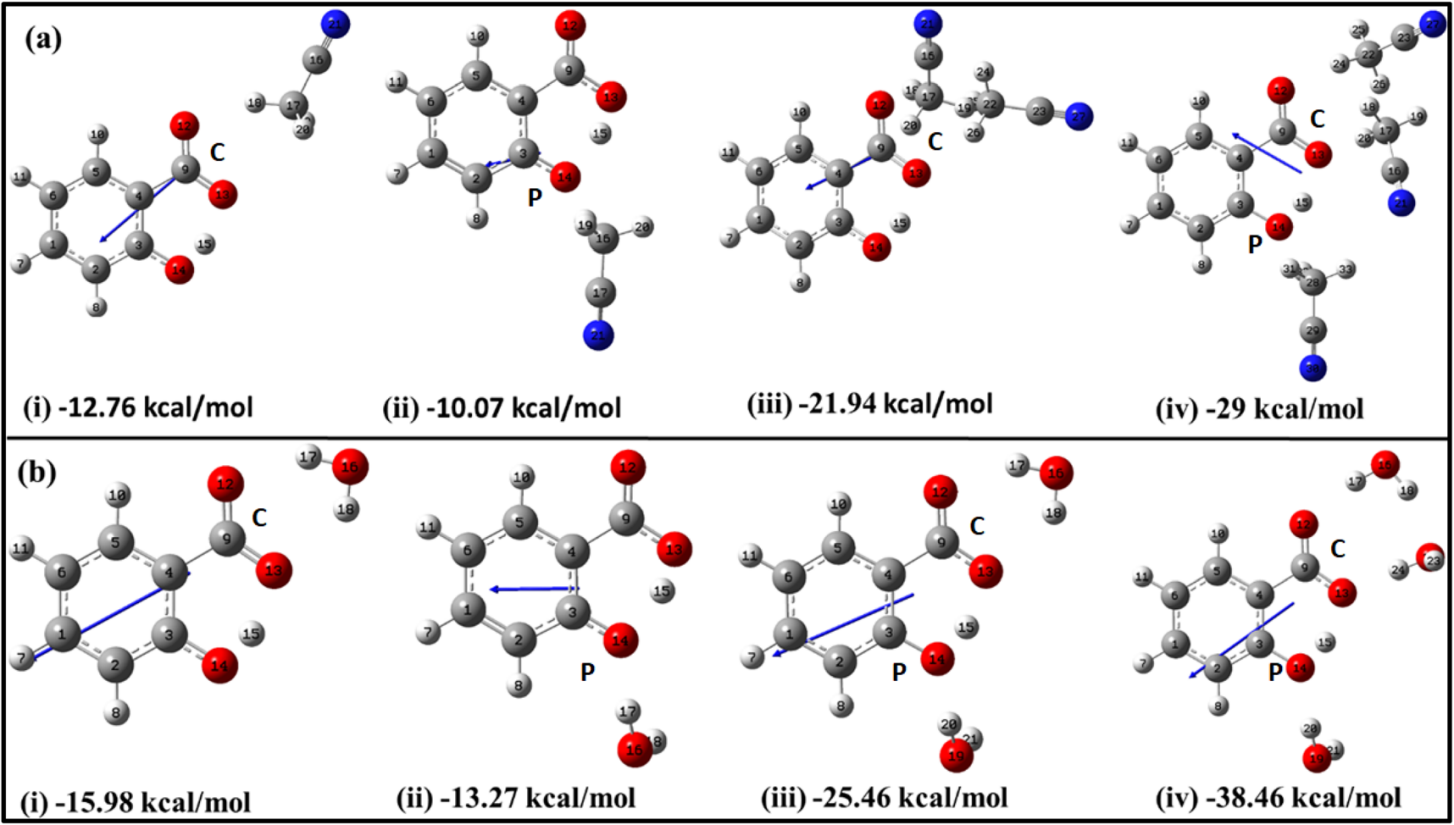
Optimized structures of salicylate anion explicitly solvated with (a) ACN and (b) water molecules at B3LYP/6-311++G(d,p) without GD3 correction, along with their stabilization energies. Single solvent molecule positioned at the (i) carboxylic position (C) (ii) phenolic position (P); (iii) two solvent molecules, and (iv) three solvent molecules.

The explicit solvation approach enables in-depth exploration of how intermolecular hydrogen bonding (I_er_MHB) of a specific solvent environment affects the solute's structure, stability, and behaviour. To understand it, electronic structure calculations have been conducted at B3LYP/6-311++G(d,p) without GD3 correction using an explicit solvent approach by placing solvent molecules in the vicinity of functional groups of salicylate anion at different positions. The optimized geometries of salicylate anion explicitly solvated with different ACN and water molecules along with their corresponding dipole moment vectors are depicted in Fig. 2a and b.

The initial insights into the stability of salicylate anion molecules in the molecular environment of ACN/water solvent molecules in the ground state were extracted from the analysis of these optimized geometries of salicylate anion moiety. During the optimization process, different stable geometries are obtained depending on the arrangement of solvent molecules relative to specific functional groups. When a single ACN molecule was positioned near either the carboxylate group or the phenolic group, optimized geometries were obtained for both placements, with the former placement being more stable (Fig. 2a(i) and (ii)). When ACN molecules were placed in proximity to both functional groups and the geometry was optimized, the ACN molecule near the phenolic position reorients itself towards the carboxyl position, resulting in symmetrical configurations concerning the carboxyl group (Fig. 2a(iii)). Furthermore, introducing a third ACN molecule near the phenolic position yielded an additional stable structure, as depicted in Fig. 2a(iv).

Similarly, the placement of a single water molecule near either the carboxylic or phenolic group also results in stable geometries with the former placement being more stable (Fig. 2b(i) and b(ii)). In contrast to the behaviour observed with ACN molecules, the introduction of two water molecules, each near a functional group, results in a stable configuration, as illustrated in Fig. 2b(iii). Further stabilization was achieved by introducing a third water molecule, which positions the water molecules near each oxygen atom, thereby yielding a stable structure.

As the number of solvent molecules increases, the direction of the dipole moment vector changes, indicating a shift in the overall polarity. Water solvated salicylate anion molecules exhibit a greater dipole moment vector compared to when it is solvated with ACN. Also, the dipole moment of the *keto* form is smaller than the *enol* form in all cases. The dipole moments of each optimized geometry have been tabulated in Table 2 along with calculated stabilization energies Δ*E*_stab_, and IMHB strength of salicylate anion as a function of the number of solvent molecules. The stabilization energies Δ*E*_stab_ of salicylate anion–solvent complexes have been calculated as follows:^42^
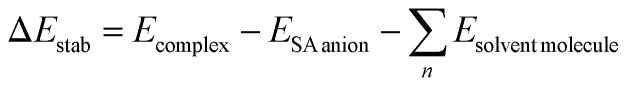


**Table 2:** Dipole moment, stabilization energies and IMHB strengths for *enol* and *keto* forms of salicylate anion explicitly solvated with the increasing number of ACN and water molecules^a^

Salicylate anion		Dipole moment (Debye)		Δ*E*_stab_ (kcal mol^−1^)		Δ*E*_IMHB_ (kcal mol^−1^)	
Number	Species	ACN	H_2_O	ACN	H_2_O	ACN	H_2_O
1(C)	*Enol*	5.941	8.375	−12.76	−15.98	23.44	23.19
	*Keto*	3.447	5.978	−10.79	−13.34	20.22	20.28
1(P)	*Keto*	3.424	4.323	−10.07	−13.27	15.70	16.95
2	*Enol*	5.964	7.524	−21.94	−25.46	21.43	18.84
	*Keto*	—	5.196	—	−25.69	—	18.17
3	*Enol*	5.271	7.380	−29.00	−38.46	16.81	16.55
	*Keto*	2.974	4.992	−26.75	−36.34	15.08	12.14

a C: carboxylate position, P: phenolic position.

Table 2 reflects the increasing stability of the salicylate anion with an increasing number of solvent molecules. The more negative stabilization energy values indicate stronger solute–solvent interactions. Notably, water molecules exhibit a greater stabilizing effect on the salicylate anion compared to ACN molecules. Further analysis reveals intriguing differences between IMHB strengths of the *enol* and *keto* forms. The analysis discloses that in the *enol* form, the IMHB strength is higher in ACN compared to water, regardless of the number of solvent molecules involved. However, in the case of the *keto* form, it is observed that the IMHB strength is initially lower in ACN as compared to water when considering one solvent molecule but becomes greater in ACN when three solvent molecules are present.

The overall optimization results reveal that an increasing number of solvent molecules around the salicylate anion increases stabilization energy, indicating the likelihood of various solvated species in the ground state. Importantly, in the explicit approach, the *keto* form of the salicylate anion was found to be more stabilized in water compared to ACN. These findings provide support for the presence of multiple decay components observed experimentally,^43^ although they differ from the implicit results, where the *enol* form was found to be more stable. In contrast to the implicit case, the IMHB of the *keto* form is lower than corresponding the *enol*. These results suggest that I_er_MHB strongly influences the stabilization of the *keto* structure.

### 3.2 Potential energy surfaces and simulated electronic absorption/emission transitions

#### 3.2.1 In implicit approach

To understand the *enol–keto* tautomerization deeply, potential energy (PE) curves were plotted for salicylate anion in the ground and excited states, which provide valuable insights into the energy changes and behaviour of the system as its molecular geometry changes. The PE curves across different functionals in implicit ACN in the S_0_ state reveal that PBE0 supports only the stabilization of the *enol* form, whereas other functionals indicate stabilization of both *enol* and *keto* forms (ESI Fig. S1†). Although the predicted electronic transitions for all functionals show significant deviations from experimental results (ESI Table S8†), B3LYP provides the closest agreement with the experimental data.^33,43^ This validates our choice of B3LYP functional.

**Fig. 3:**
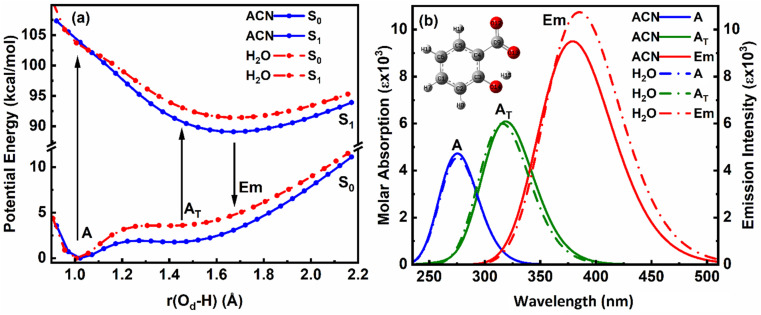
Overlapped (a) PE curves and (b) corresponding absorption–emission plot of *enol* (A) and *keto* (A_T_) structures of salicylate anion in the implicit environment of ACN and water at B3LYP/6-311++G(d,p).

The ground and excited singlet state PE curves for salicylate anion in ACN and water along with computationally simulated electronic absorption/emission transitions have been shown in Fig. 3a and b, respectively. A comprehensive summary of the geometric parameters in both solvents is presented in Table 3.

**Table 3:** Computationally calculated spectral parameters of salicylate anion in implicit ACN and water

Solvent	Species	*λ*_abs_ (nm) (*f*)	*λ*_em_ (nm) (*f*)	Δ*ν* (cm^−1^)	Dipole moment (Debye)			IMHB (kcal mol^−1^)		
					*μ*_g_	*μ*_e_	Δ*μ*	S_0_	S_1_	Δ*E*_IMHB_
ACN	A	275.35 (0.1167)	379.07 (0.2345)	9937	9.887	7.770	−2.117	13.09	8.36	−4.73
	A_T_	318.89 (0.1503)		4978	7.314		0.456	15.06		−6.7
Water	A	275.7 (0.1125)	385.15 (0.2652)	10 307	11.436	10.115	−1.321	7.98	5.76	−2.22
	A_T_	315.77 (0.1482)		5705	8.775		1.340	10.65		−4.89

The ground state (S_0_) PE profile of salicylate anion exhibits dual minima in both ACN and water (Fig. 3a). The primary *enol* form (A) has a deeper minimum due to resonance stabilization, while the tautomeric *keto* form (A_T_) has a shallower, flattened minimum. Despite both forms being stable, the *enol* form is more stable in both solvents. The forward GSIPT (A → A_T_) faces barriers of 1.917 kcal mol^−1^ in ACN and 3.578 kcal mol^−1^ in water, while the reverse GSIPT (A_T_ → A) requires much lower barriers of 0.159 kcal mol^−1^ and 0.005 kcal mol^−1^, respectively. Despite the higher IMHB strength in ACN (Table 3), the energy required to cross the barrier for *enol–keto* tautomerization in the ground state is primarily achieved through thermal energy.

When the *enol* form (A) absorbs radiation, it becomes excited *enol* (A*), which quickly converts to the *keto* form (A_T_*) in the first excited state (S_1_) *via* a barrierless ESIPT reaction. The S_1_ PE curve has a single flattened well, indicating stabilization of A_T_* in the excited state. The relaxation of this A_T_* to the ground state A_T_ results in tautomeric emission, and eventually, A_T_ converts back to A *via* reverse GSIPT. In higher excited states (S_2_ and S_3_) (ESI Fig. S2a and b†), both *enol* and *keto* forms are stable and show double-well potentials, but rapid internal conversion results in observed emission only from A_T_* in the S_1_ state. The oscillator strength increases rapidly and then gradually during the S_0_ → S_1_ transition in both solvents, with the A_T_* form having about 1.3 times the strength of the *enol* form (ESI Fig. S2c†). The excited state dipole moment decreases as *r*(O_d_-H) increases in both solvents, with the *enol* form's dipole moment decreasing and the *keto* form's increasing by 1.33 debye during the S_0_ → S_1_ transition. After adiabatic relaxation in the first excited state, the IMHB strength decreases to 8.36 kcal mol^−1^ in ACN and 5.76 kcal mol^−1^ in water (Table 3). The *keto* form's IMHB strength in the ground state is nearly double that in the excited state, indicating significant stabilization. Hence, dual minima for *enol* and *keto* forms are observed in the ground state PE profile.

In ACN, both *enol* (A) and *keto* (A_T_) forms of salicylate anion absorb at 275.35 nm and 318.89 nm, with oscillator strengths of 0.1167 and 0.1503, respectively (Fig. 3b). Both geometries emit at 379.07 nm with an oscillator strength of 0.2345. In water, the absorption maxima for *enol* and *keto* structures appear at 275.7 nm and 315.77 nm, with oscillator strengths of 0.1125 and 0.1482, respectively. However, both structures emit at 385.15 nm with 0.2652 oscillator strength in water. In both the solvents, the absorption/emission transitions are of π → π* character. Thus, the absorption maximum of the *enol* structure is nearly the same in both solvents with nearly equal transition probability. The absorption maximum of *keto* form is slightly blue-shifted by ∼3 nm in water with a lower transition probability. The emission maximum is significantly red-shifted in water by 6 nm with higher transition probability, resulting in a large stoke shift of approximately ∼10 000 cm^−1^. The slightly higher oscillator strength of the emission transition in water compared to ACN suggests that the polarity of the solvent is enhancing the emission intensity.

On comparing our quantum computational findings with the reported experimental results,^21,33,34^ it is evident that both the *enol* and *keto* forms of salicylate anion are stable in ACN and water. However, ∼5.32% of salicylate anions are present in *keto* forms in ACN, while only ∼0.26% of molecules are in *keto* form in water. This contrasts with recent experimental studies,^33^ which supported the stability of only the *enol* form in water. The *enol* form exhibits greater stability than the *keto* form in the ground state. The barrier height for interconversion between the two forms in the ground state is approximately twice as high in water compared to ACN, indicating a lower presence of the *keto* form in water. The oscillator strength of the *keto* form for the S_0_ → S_1_ transition is greater than that of the *enol* form, yet due to the higher stability of the *enol* form, its absorption appears more prominently in experimental results. In water, the emission spectra display a red-shift accompanied by broadening, which can be attributed to the increased dipole moment and polarizability of the salicylate anion in this solvent. However, it is noteworthy that while computationally simulated emission results indicate a higher S_1_ → S_0_ transition probability in water, experimental observations reveal fluorescence quenching, suggesting additional factors contributing to the decrease in fluorescence intensity.

#### 3.2.2 In explicit solvation approach

**Fig. 4:**
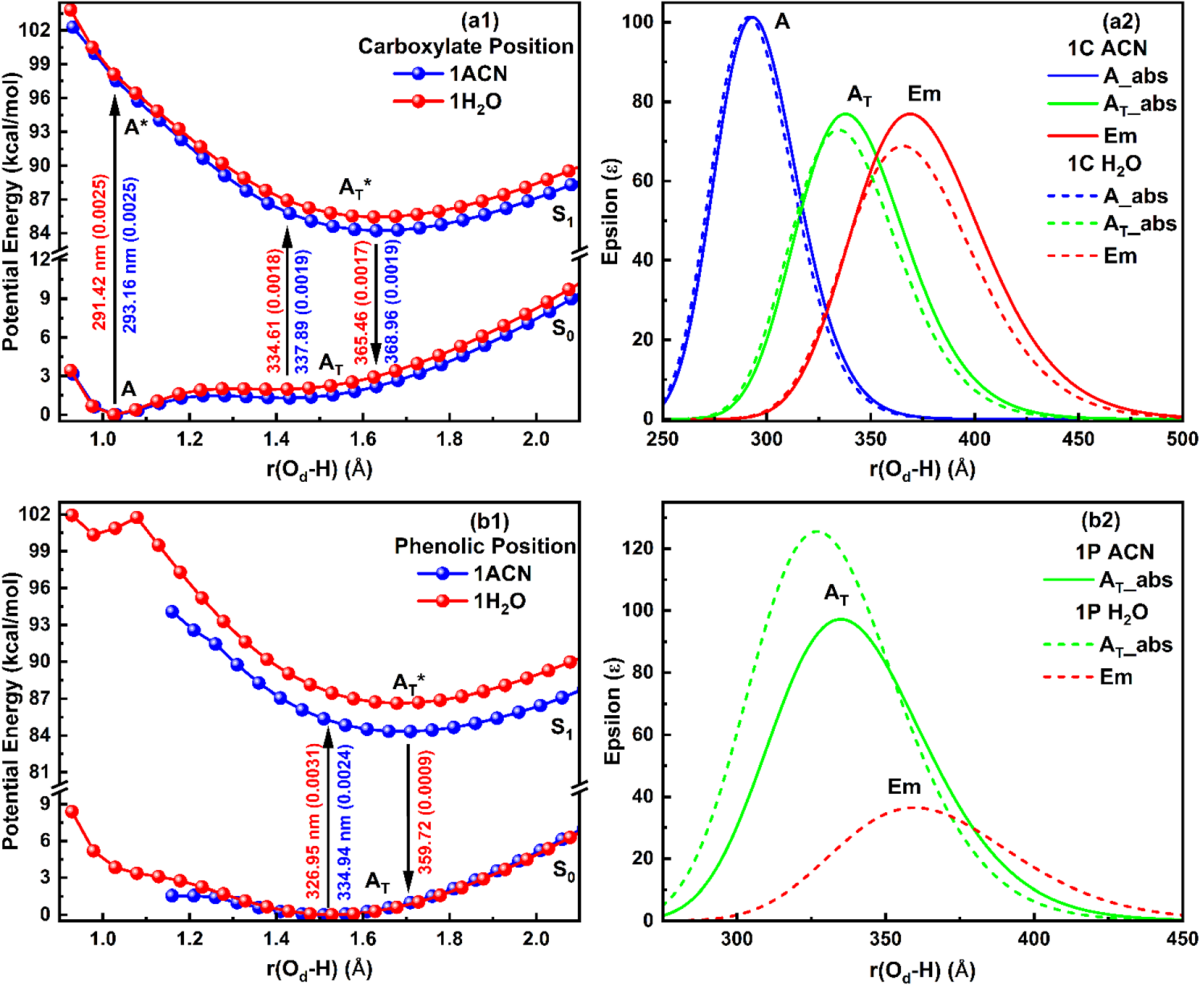
Potential energy curve (Left) and corresponding absorption-emission spectra (right) for the salicylate anion molecule explicitly solvated with one solvent molecule at (a) carboxylate (b) phenolic functional group.

Further, to understand the effect of explicit solvated ACN and water molecules on the photophysical parameters of salicylate anion, the PE profile and absorption-emission spectral transitions were computationally simulated, and the overlapped ground and first excited state PE curves, along with overlapped simulated absorption/emission spectra are displayed in Fig. 4. The calculated IMHB, dipole moment, absorption/emission maxima and corresponding oscillator strength are tabulated in Table 4.

**Table 4:** Computationally simulated spectral parameters of salicylate anion in explicit solvent^a^

Solvent	Position	Species	*λ*_abs_ (nm)	*f*	*λ*_em_ (nm)	*f*	Stokes shift	Dipole moment, (D)		
			S_0_ → S_1_	S_0_ → S_1_	S_1_ → S_0_	S_1_ → S_0_	Δ*ν̄* (cm^−1^)	*μ*_g_	*μ*_e_	Δ*μ*
ACN	1(C)	A	293.16	0.0025	368.96	0.0019	7008	5.941	11.970	6.029
		A_T_	337.89	0.0019			2492	3.447		8.523
	1(P)	A_T_	334.94	0.0024	—	—	—	3.424	—	—
	2	A	280.47	0.0028	—	—	—	5.964	—	—
	3	A	274.65	0.0904	—	—	9471	5.271	—	1.404
		A_T_	318.51	0.1358	371.21	0.1186	4443	2.974	6.675	3.701
Water	1(C)	A	291.42	0.0025	—	—	6952	8.375	—	−3.932
		A_T_	334.61	0.0018	365.46	0.0017	2523	5.979	4.443	−1.536
	1(P)	A_T_	326.95	0.0031	359.72^b^	0.0009	2786^b^	4.323	—	—
	2	A	274.83	0.0038	—	—	8585	7.524	—	−1.745
		A_T_	315.06	0.1234	359.7	0.1175	3939	5.196	5.779	0.583
	3	A	270.67	0.0193	—	—	9425	7.380	—	−1.719
		A_T_	310.97	0.1222	363.37	0.1187	4637	4.992	5.661	0.669

a C: carboxylate site, P: phenolic site.

b Freezed.

When a single ACN or water molecule is positioned in the vicinity of the carboxylate functional group, the ground state PE curve exhibits dual minima corresponding to the *enol* (A) and *keto* (A_T_) structures, with *enol* form more than stable than *keto* form, as shown in Fig. 4a1. In contrast, the excited state PE curve displays a single well corresponding to the *keto* form (A_T_*). Strikingly, both the absorption and emission spectra show a blue shift in water compared to ACN (Fig. 4a2). The S_0_ → S_1_ transition probability is the same for the *enol* form in ACN and water, while it decreases for the *keto* form in water. Also, the S_1_ → S_0_ transition probability for *keto* form is less in water. The barrier potential for the *enol–keto* transformation is calculated to be 1.477 kcal mol^−1^ for ACN and 2.018 kcal mol^−1^ for water in the ground state. The dipole moment (*μ*) is found to increase in ACN, while decreases in water in the excited state for both *enol* and *keto* forms, as presented in Table 4.

An intriguing observation emerges when the solvent molecule is positioned near the phenolic group of the salicylate anion. In this configuration, the PE profile exhibits a single well corresponding to the tautomeric *keto* form in both the ground and excited states, as depicted in Fig. 4b1. Furthermore, the absorption maximum of the *keto* form is blue-shifted, accompanied by an increased transition probability (*f*) in water compared to ACN, as illustrated in Fig. 4b2. This finding suggests that I_er_MHB facilitates the translocation of the proton from the phenolic oxygen (O_a_) to the carboxylate oxygen (O_d_) in the ground state through GSIPT. Notably, emission corresponding to the *keto* form in water is observed at 359.72 nm (in frozen state), while optimization did not converge in ACN in an excited state, therefore, emission couldn't be predicted.

**Fig. 5:**
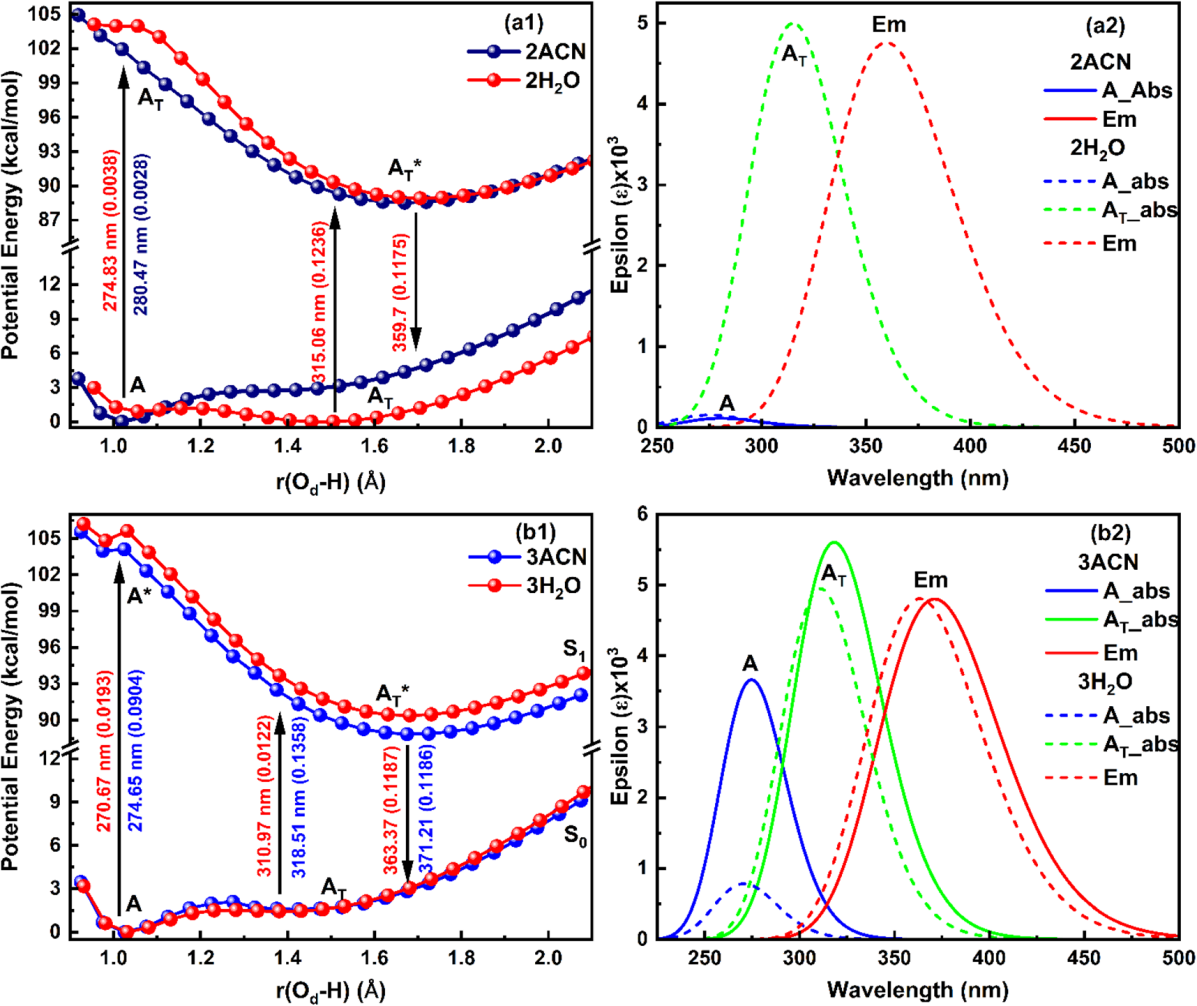
Potential energy curve (Left) and corresponding absorption-emission spectra (right) for the salicylate anion molecule explicitly solvated with (a) two solvent molecules near both functional groups, and (b) three solvent molecules near all oxygen atoms of functional groups.

In the presence of two ACN molecules, the ground state PE exhibits a single minimum corresponding to the *enol* form. This *enol* form shows S_0_ → S_1_ transition at 280.47 nm with oscillator strength 0.0028, however, no convergence was found in the excited state. On the other hand, a dual well PE curve is observed in the ground state in the presence of two water molecules, with the *keto* form being more stable than *enol* (Fig. 5a1). The simulated absorption and emission spectra of salicylate anion with two molecules of ACN/water are displayed in Fig. 5a2. The absorption maxima for both *enol* and *keto* forms in water occur at 274.83 nm and 315.06 nm respectively. The oscillator strength of the *keto* form (0.1236) is found nearly 32 times higher than the *enol* form (0.0038). In the excited state, only the *keto* structure remains stable. Consequently, the excited *enol* form relaxes to the *keto* structure through ESIPT and emits at 359.7 nm. The Stokes shift corresponding to the *enol–keto* tautomerization is approximately 8585 cm^−1^ (ESIPT emission), which is greater than the Stokes shift of the *keto* form at approximately 3939 cm^−1^ (normal emission). Therefore, the *enol* form exhibits a blue-shifted absorption maximum in the presence of water, accompanied by a higher transition probability compared to ACN, and emits in the *keto* region.

Further, when three ACN/water molecules are positioned near each oxygen atom, the PE curves in the ground state again exhibit dual minima with *enol* form more stable than *keto* form in the presence of both ACN and water (Fig. 5b1). Interestingly, the ground state PE curves appear to overlap with slight differences. The barrier potential for the *enol* to *keto* transformation is 2.09 kcal mol^−1^ in ACN and decreases to 1.53 kcal mol^−1^ in water. Salicylate anion shows a blue-shifted absorption maxima in water than ACN for both *enol* and *keto* forms. In water, there is a drastic decrease in the transition probability (*f*) for the *enol* form compared to the *keto* form, as shown in Fig. 5b2. Moreover, the absorption maxima for the *enol* and *keto* structures of the salicylate anion solvated with three water molecules exhibit a more significant blue shift compared to when only one water molecule is positioned near the carboxylate oxygen. Additionally, the oscillator strength increases significantly in the presence of multiple water molecules. In the excited state, the *enol* form undergoes relaxation to the *keto* form through the ESIPT process and emits at 363.37 nm, resulting in a Stokes shift of approximately 9425 cm^−1^, which is greater than the Stokes shift of the *keto* form (4637 cm^−1^). Furthermore, the Stokes shift for the salicylate anion solvated with three water molecules is greater for both the *enol* and *keto* forms compared to the species with single or double water molecules. A successive red-shift appears in emission spectra on increasing the ACN/water molecules near to salicylate anion, as shown in ESI Fig. S3.†

These findings reveal a higher barrier height for *enol–keto* tautomerization in water when using implicit solvation, along with a red-shifted fluorescence and increased transition probability. This indicates a lesser presence of the *keto* form in water compared to acetonitrile (ACN). However, in explicit solvation, the PE curves show a similar trend with one solvent molecule, but the curves overlap with three solvent molecules. Interestingly, the explicit solvation model predicts blue-shifted absorption and emission spectra in water compared to ACN with increased transition probabilities, contrary to the implicit solvation model. These results did not match well with the reported experimental observation^34^ except for a red shift in emission spectra in water. This implies that both solvent polarity parameters and hydrogen bonding of solvent environment are not the root cause of fluorescence quenching of salicylate anion in water indicating the presence of some other pathways for energy dissipation.

### 3.3 Molecular electrostatic potential (MEP) and natural charges analysis

MEP and Natural charge distribution analyses were conducted to investigate charge distribution and electron density variation between the *enol* and *keto* structures of the salicylate anion solvated implicitly and explicitly with ACN and water molecules. The MEP plot provides a visual representation of the electrostatic potential surrounding the molecule. The blue regions in the MEP plot indicate areas of low electron density or regions with positive electrostatic potential. On the other hand, red regions represent areas of high electron density or regions with a negative electrostatic potential. These colour variations provide visual cues for identifying regions of electron-rich and electron-deficient regions within the molecule. Analysis of Charge distribution is performed *via* Natural Population Analysis (NPA) along with Mulliken Population Analysis (MPA) which provides insights into changes in electron density in the ground and excited state.

#### 3.3.1 Implicit MEP plot analysis and NPA

**Fig. 6:**
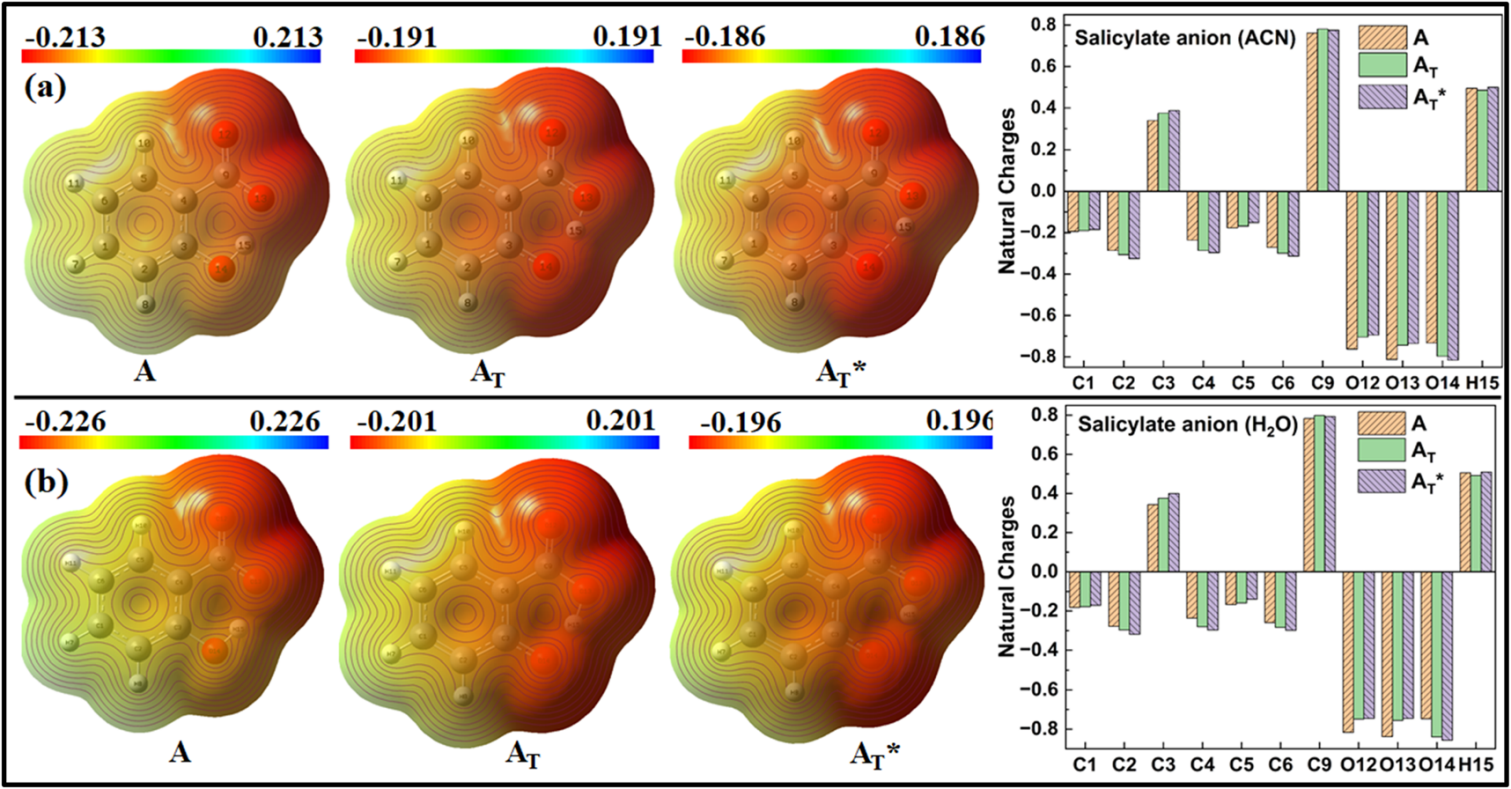
MEP plot and Natural charge distribution plot of salicylate anion in implicit (a) ACN (b) H_2_O.

Fig. 6 shows the MEP plot and charge distribution plot of salicylate anion using NPA in implicitly solvated salicylate anion in the dielectric environment of ACN and water. All the carbons atoms show natural negative charge, except C3. In both solvents, the *enol* structure's hydroxyl group exhibited lower negative charge density than the carboxylic group in the ground state. Conversely, the ground and excited states of the *keto* structure showed increased charge density near the phenolic oxygen (O14). This resulted in a decrease in the *keto* structure's dipole moment compared to the *enol* structure. The range of charge density was larger in the *enol* structure's ground state and smaller in both the ground and excited states of the *keto* structure as concluded by NPA.

In contrast, Mulliken analysis (ESI Fig. S4†) reveals that in ACN, proton transfer causes a reversal in polarity of the charge density at C2 and C9 atoms, while the magnitude of charge density increases on other carbon atoms. Modest changes in charge density were observed on the oxygen atoms, with a more pronounced effect in the excited state. In water, the charge density at atoms C1, C2, and C9 reverses polarity in the excited state, while on other atoms, the charge density only changes in magnitude. Changing the dielectric environment from ACN to water has little impact on the *enol* form but led to considerable changes at all carbon atoms of the *keto* form. In water, charge density reduction was observed in *keto* form at C1, C3, C4, and C5 atoms, while an increase was observed at C6 atom. Surprisingly, the charge density at C2 reversed. In the excited state, modest variations in charge density were observed when transitioning from ACN to water.

Overall, these analyses elucidated the variations in charge density, dipole moment, and polarity between the *enol* and *keto* structures of the salicylate anion in different solvents, emphasizing the solvent's influence on the molecule's electronic properties.

#### 3.3.2 Explicit MEP plot analysis and NPA

**Fig. 7:**
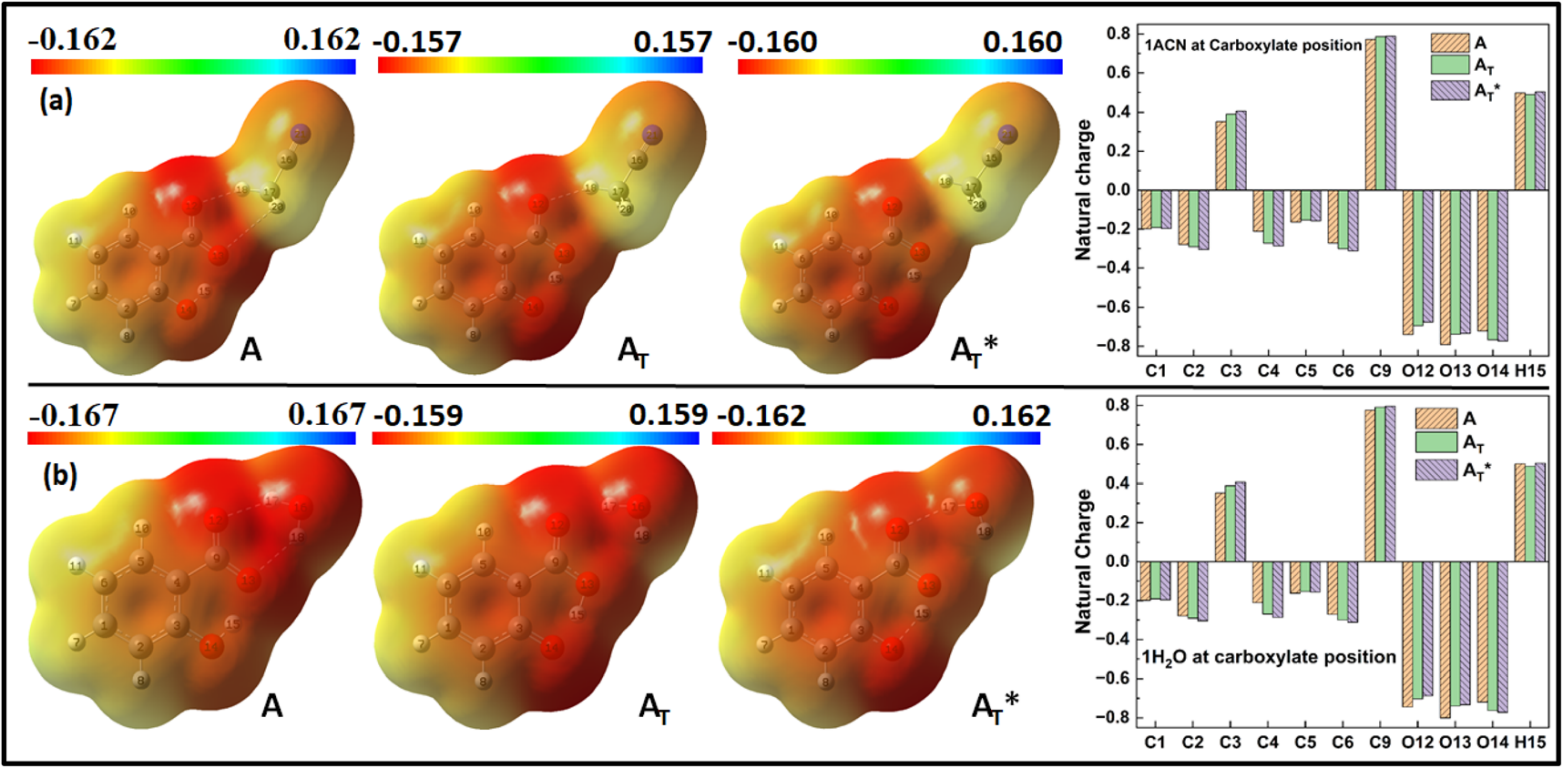
MEP plot (left) and Natural charge distribution (right) of salicylate anion molecule in excited state explicitly solvated with a single molecule of (a) ACN and (b) water placed at carboxylic position.

Fig. 7 illustrates the MEP plot and natural charge distribution for the salicylate anion when explicitly solvated by a single solvent molecule at the carboxylic position. Since the salicylate anion itself carries a negative charge, the MEP plot depicts the expected negative potential around the molecule. The negative charge density is concentrated near the oxygen atoms, as evident in Fig. 7a and b for both ACN and water molecules in the ground state. The *keto* form exhibits slightly higher negative charge density near the O14 atom (donor) than the ground state's *enol* form. In terms of variations, the *enol* form displays greater changes in charge density compared to the *keto* form in both ground and excited states for both ACN and water molecules, similar to implicit case.

The Natural charge distribution analysis reveals that carbon atoms directly attached with oxygen atoms (C3 and C9) acquire positive charges while other carbons are negative. Subtle changes in charge distribution among the atoms of the *enol* and *keto* forms were observed in the S_0_ state. In the excited state with ACN (Fig. 7a), there is an increase in charge density at C3 while a decrease at C4 is observed. All the other carbon atoms exhibit a reversal in charge density. However, when solvated with a water molecule (Fig. 7b), minimal variations in charge density are observed in the excited state.

**Fig. 8:**
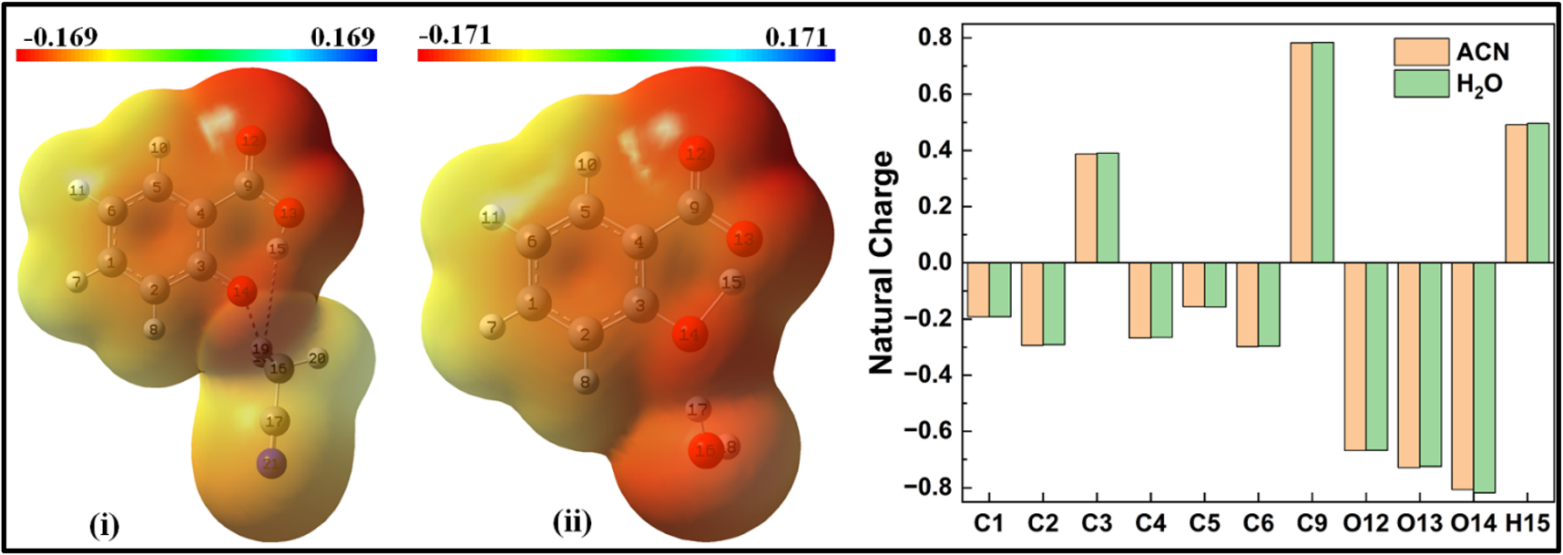
MEP and Natural charge distribution plots for salicylate anion explicitly solvated with single (i) ACN (ii) water molecule, placed at the phenolic position.

When a solvent molecule is placed at the phenolic position (Fig. 8), the MEP plot of the salicylate anion shows a slightly broader range of charge density variations in water compared to ACN. No significant differences are observed in the ground-state natural charge distribution between the ACN and water solvents. Contrary to this, Mulliken charge distribution exhibits changes at all carbons atoms except C3 and C6 atoms (ESI Fig. S5†).

### 3.4 Natural bond orbital (NBO) analysis

The NBO analysis provides valuable insights into the variations in charge densities within proton donor and acceptor species, as well as in bonding and antibonding orbitals, and thus, sheds light on the influence of these interactions on the overall stability of the optimized geometries.^52^ This NBO analysis is aimed at understanding the intra- and intermolecular hydrogen bonding interactions for the salicylate anion in both solvents. The formation of hydrogen bonds is primarily attributed to the transfer of charge from the lone pair *i.e.* the non-bonding electron orbitals (n or LP) of proton acceptor (O_a_) to the antibonding (σ*) orbital of proton donor (O_d_),^54^ accompanied by elongation and contraction of the H⋯O bond. The second-order perturbation stabilization energy (Δ*E*_ij_^(2)^) associated with the delocalization between the donor (i) and acceptor (*j*) is calculated using the following equation:
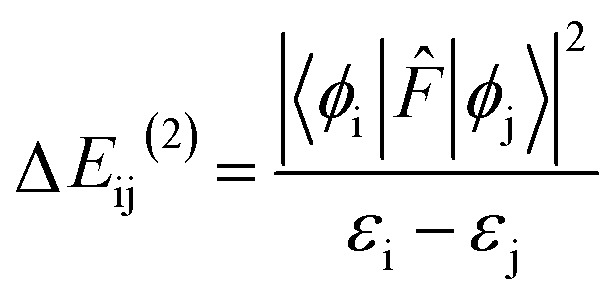
where *F̂* is the fock operator, and *ε*_i_ and *ε*_j_ are energy eigenvalues of the donor (*ϕ*_i_) and acceptor (*ϕ*_j_) molecular orbitals, respectively.

#### 3.4.1 Implicit solvation NBO analysis

**Fig. 9:**
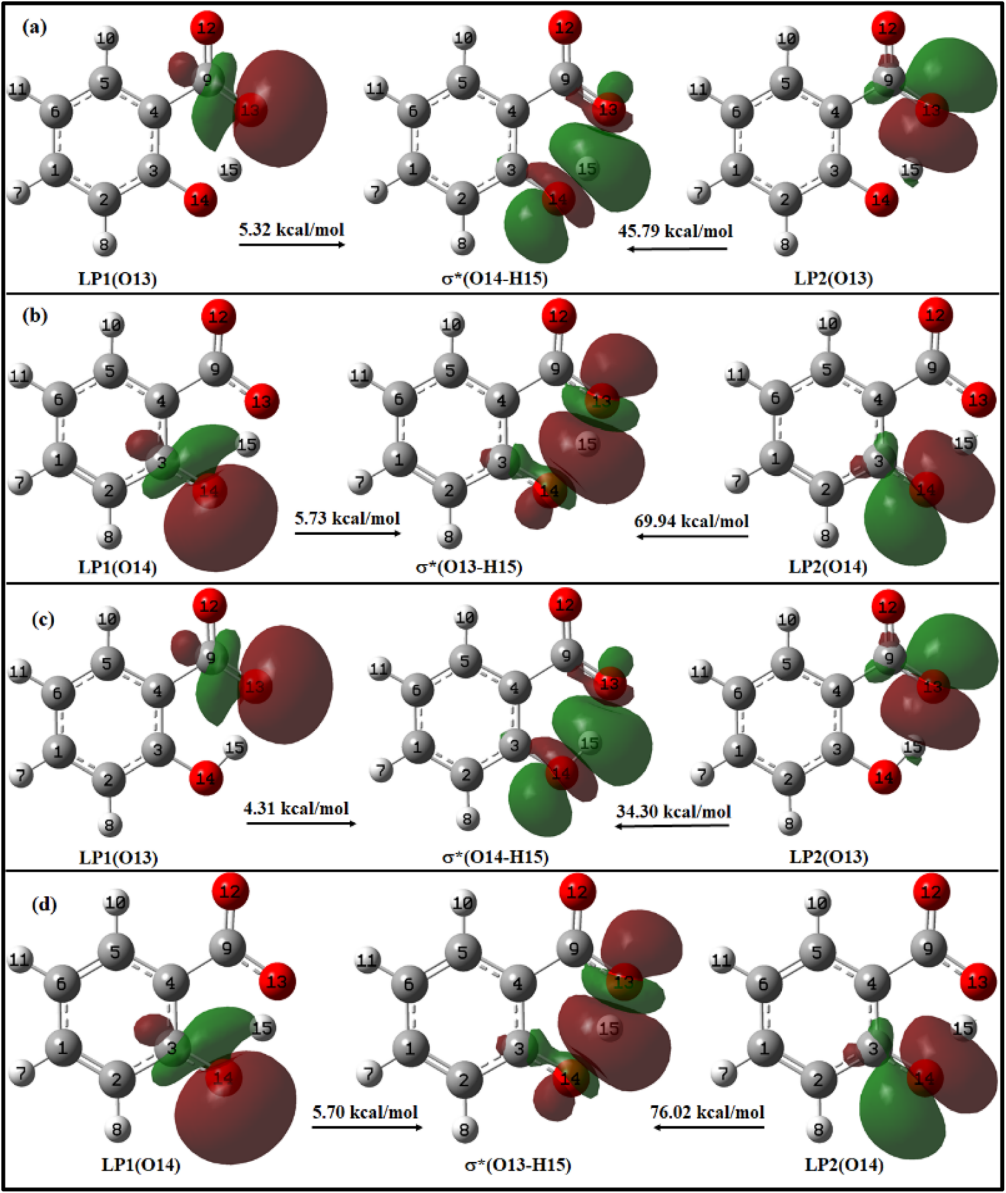
Charge delocalization from lone pair (LP) orbital to σ* antibonding orbital in (a) *enol* and (b) *keto* form of salicylate anion in ACN; and (c) *enol* and (d) *keto* form of salicylate anion in water, in the ground state.

The summarized results of NBO analysis for the *enol* and *keto* forms of salicylate anion in the implicit environment of both solvents are presented in ESI Table S9† and the charge delocalization from lone pair (LP) orbitals to σ* antibonding orbitals is shown in Fig. 9.

For *enol* form, the energy corresponding to LP1(O13) → σ*(O14–H15) and LP2(O13) → σ*(O14–H15) interactions are 5.32 kcal mol^−1^ and 45.79 kcal mol^−1^ in ACN (Fig. 9a), and 4.31 kcal mol^−1^ and 34.30 kcal mol^−1^ in water (Fig. 9c). Thus, a total stabilization energy of 51.11 kcal mol^−1^ in ACN and 38.61 kcal mol^−1^ in water is involved in LP(O13) → σ*(O14–H15) interaction. Considering the *keto* form, the lone pair orbital LP1 of O14 oxygen atom is aligned about the bond axis C3–O14 while lone pair orbital LP2 of O14 is aligned perpendicular to bond axis C3–O13. However, σ* antibonding orbital is aligned about the bond axis O13–H15. The energy corresponding to LP1(O14) → σ*(O13–H15) and LP2(O14) → σ*(O13–H15) interactions are 5.73 kcal mol^−1^ and 69.94 kcal mol^−1^ in ACN (Fig. 9b), and 5.70 kcal mol^−1^ and 76.02 kcal mol^−1^ in water (Fig. 9d). Other relevant LP → σ* interactions are tabulated in ESI Table S9.†

From these observations, it can be deduced that the *enol* form exhibits relatively lower stabilization energies and weaker LP → σ* interactions compared to the *keto* form in both ACN and water. The *keto* form demonstrates higher stabilization energies and stronger LP → σ* interactions, suggesting a more favourable hydrogen bonding environment in both solvents. This information suggests that IMHB strength is greater for *keto* form than *enol* form which is well–correlated with the IMHB strength reported in Table 1. Further, the LP → σ* interactions are found to be stronger in ACN than in water for the *enol* form, while in the case of the *keto* form, they are stronger in water. The overall IMHB strength is greater in ACN for both the *enol* and *keto* forms compared to water (Table 1). These results provide insights into the stability and hydrogen bonding characteristics of the *enol* and *keto* forms of salicylate anion in different solvents.

#### 3.4.2 Explicit solvation NBO analysis

**Fig. 10:**
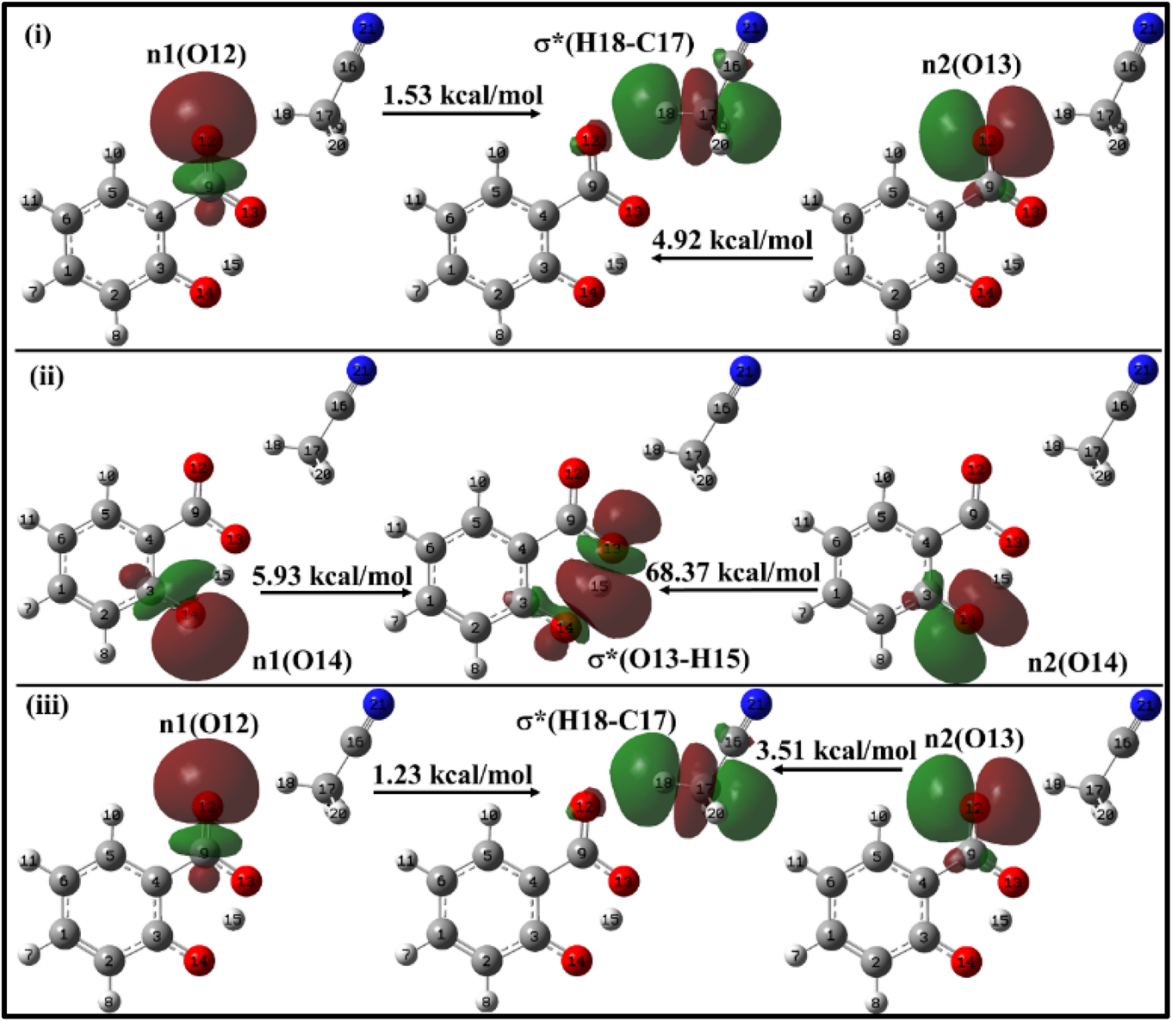
Delocalization of charge (i) from *enol* form to ACN molecule; (ii) within *keto* form of salicylate anion, (iii) from *keto* form to ACN molecule situated near carboxylate group.

When the salicylate anion is solvated by ACN or water molecules at different positions, intra- and intermolecular n → σ* interactions occur, leading to the formation of hydrogen bonds. Fig. 10 illustrates the IMHB arising due to n → σ* interactions within salicylate anion moiety, and I_er_MHB arising between salicylate anion and ACN molecule near the carboxylate position, respectively.

In the *enol* form (Fig. 10i), the lone pair n1 of the O12 oxygen atom and σ*(C17–H18) bond of ACN molecule are nearly perpendicular to each other; interactions result in a stabilization energy of 1.53 kcal mol^−1^. The alignment of the second lone pair, n2, of the O12 oxygen atom, coincides with the direction of the σ*(H18–C17) bond, and the interaction between them leads to a stabilization energy of approximately 4.92 kcal mol^−1^. These interactions contribute to a total stabilization energy of 6.45 kcal mol^−1^. However, as the H15 atom is not bonded to O14 and acts as a dissociated unit, no n → σ* intramolecular interactions are present. In the *keto* form (Fig. 10(iii)), the intramolecular interaction n(O14) → σ*(O13–H15) results in the stabilization of 74.3 kcal mol^−1^ (5.93 kcal mol^−1^ and 68.37 kcal mol^−1^), while the intermolecular interaction n(O12) → σ*(C17–H18) energy of 4.74 kcal mol^−1^ (1.23 kcal mol^−1^ and 3.51 kcal mol^−1^).

**Fig. 11:**
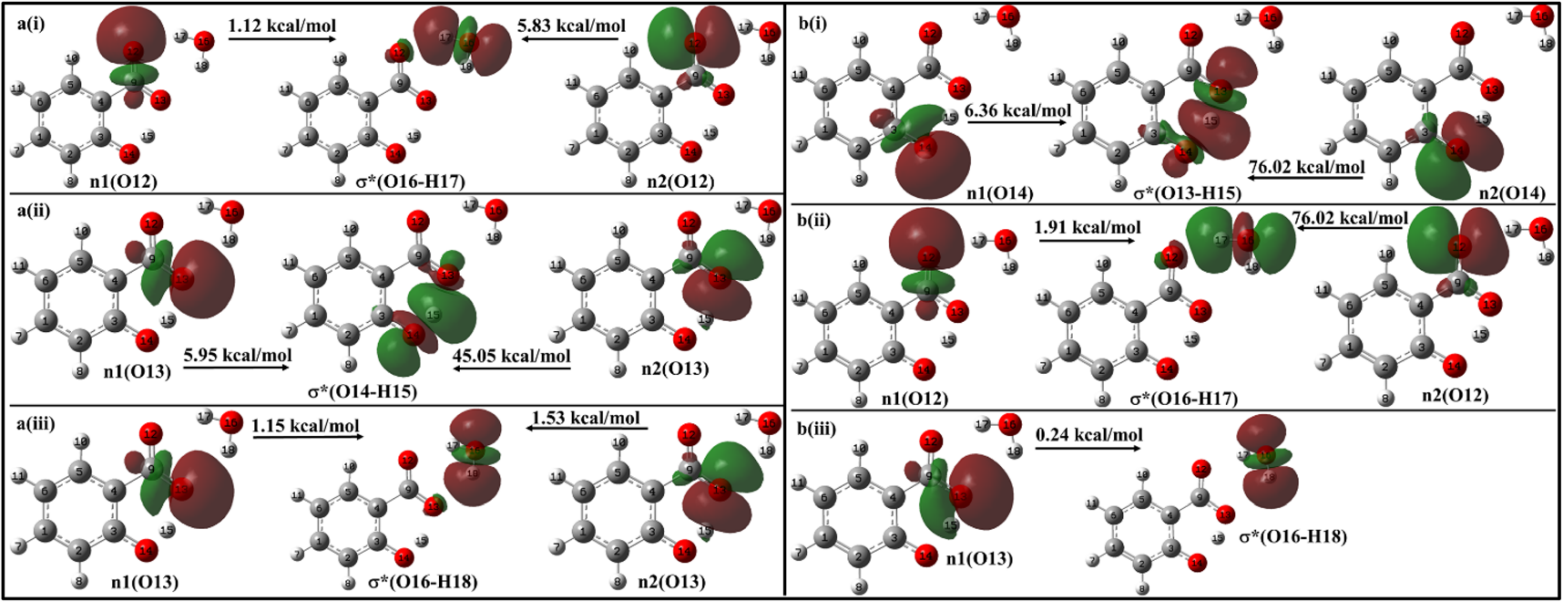
Delocalization of charge from the lone pair orbital of (a) *enol* and (b) *keto* forms of salicylate anion moiety to σ* molecular orbital of H_2_O molecule situated near carboxylate group.

With a single water molecule in the vicinity of the carboxylic group of the *enol* form (Fig. 11a), the first lone pair n1 of O12 oxygen atom of the carboxylate group is aligned along the C9–O12 bond while the second lone pair n2 of O12 is aligned in the direction of H17–O16 bond. Both the lone pairs interact with σ*(O16–H17) with stabilization energies of 1.12 kcal mol^−1^ and 5.83 kcal mol^−1^, respectively, resulting in a net stabilization of 6.95 kcal mol^−1^ (Fig. 11a(i)). Similarly, the lone pairs n1 of O13 oxygen atom interacts with intramolecular σ*(O14–H15) with 51 kcal mol^−1^ stabilization energy (5.95 kcal mol^−1^ due to n1 and 45.05 kcal mol^−1^ due to n2) (Fig. 10a(ii)), and with intermolecular σ*(O16–H18) with 2.68 kcal mol^−1^ (Fig. 11a(iii)).

In the *keto* form with a single water molecule in the vicinity of the carboxylic group (Fig. 11b), the intramolecular n(O14) → σ*(O13–H15) interactions cause stabilization of 82.38 kcal mol^−1^ (6.36 kcal mol^−1^ due to n1 and 76.02 kcal mol^−1^ due to n2) (Fig. 11b(i)). The lone pairs of O12 atoms interact with σ*(O16–H17) by a net energy of 77.93 kcal mol^−1^ (Fig. 11b(ii)) while n(O13) interacts negligibly with σ*(O16–H18) with 0.24 kcal mol^−1^ (Fig. 11b(iii)). The solvent molecule at the carboxylate region makes hydrogen bond with the carboxylate oxygen. This causes a weakening of IMHB strength. Therefore, the *enol* form is energetically favoured by the *keto* form.

**Fig. 12:**
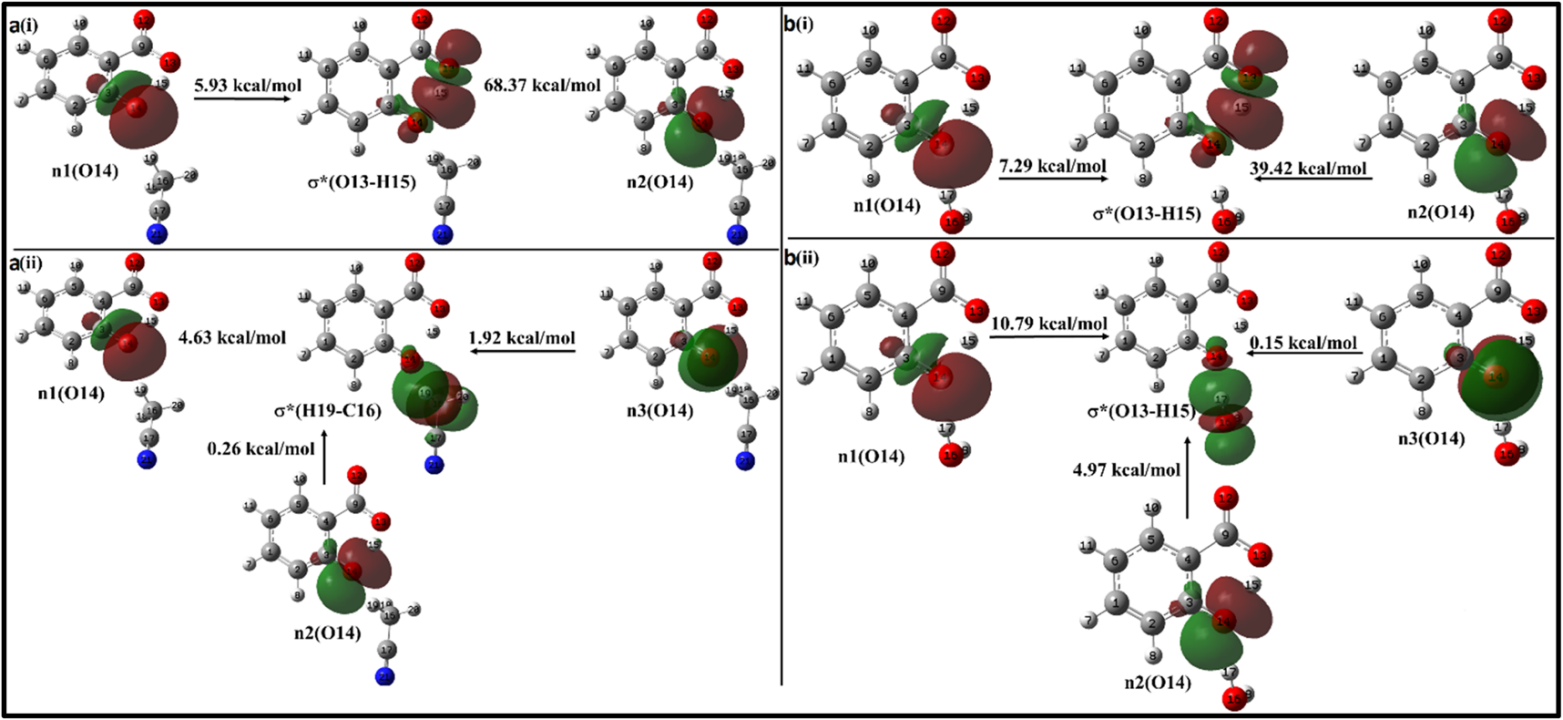
Delocalization of charge from the lone pair orbital of salicylate anion moiety to σ* molecular orbital of (a) ACN (b) H_2_O molecule situated near the phenolic group. (i) Intramolecular interaction, (ii) intermolecular interaction.

Moving on to Fig. 12a, where the ACN molecule is positioned near the phenolic position, intra- and intermolecular HB interactions occur. Intramolecular n1(O14) → σ*(O13–H15) n2(O14) → σ*(O13–H15) interactions contribute to stabilization energies of 5.93 kcal mol^−1^ and 68.37 kcal mol^−1^ (Fig. 12a(i)), resulting from the perpendicular and parallel alignment of lone pair orbitals with respect to σ*(O13–H15). The intermolecular energetic interactions involving the lone pairs n1, n2, n3, and σ*(H19–C16) are measured at 4.63, 0.26, and 1.92 kcal mol^−1^, respectively, leading to a cumulative stabilization of 6.81 kcal mol^−1^ (Fig. 12a(ii)).

The presence of water molecules at the salicylate anion's phenolic position leads to inter- and intramolecular n → σ* interactions (Fig. 12b). The intramolecular interactions n1(O14) → σ*(O13–H15) and n2(O14) → σ*(O13–H15) result in a net stabilization of 46.71 kcal mol^−1^ (7.29 kcal mol^−1^ and 39.42 kcal mol^−1^, respectively) as shown in Fig. 12b(i). However, three lone pairs n1, n2 and n3 of O14 oxygen atom interact by I_er_MHB interactions *via* 10.79 kcal mol^−1^, 4.97 kcal mol^−1^ and 0.15 kcal mol^−1^ stabilization energies (Fig. 11b(ii)). Due to these interactions, the *keto* form becomes more stable than its *enol* form. Thus, the solvent molecule at the phenolic position makes hydrogen bond with phenolic oxygen, weakening the O_d_-H bond strength. So, the phenolic hydrogen is easily attracted by the carboxylate oxygen along IMHB. Hence, in this case, the *keto* form is energetically favoured.

In salicylate anion optimized with two ACN molecules, both present near the carboxylic group, the intramolecular interaction n(O13) → σ*(O14–H15) causes stabilization of 46.66 kcal mol^−1^, while intermolecular interactions n(O13) → σ*(C17–H18) and n(O13) → σ*(C22–H26) lead to stabilization of 3.84 kcal mol^−1^ and 4.23 kcal mol^−1^ (ESI Fig. S6†). With water molecules at both positions, the *keto* form is more stable than the *enol* form, meaning I_er_MHB at the phenolic position dominates over the carboxylate position, resulting in ease in the proton transfer process (ESI Fig. S7†). Therefore, a competition between I_er_MHB and IMHB occurs.

On adding one more solvent molecule (*i.e.* presence of three solvent molecules), the explicit solvent molecules near carboxylate oxygen atoms interact strongly through I_er_MHB as compared to the phenolic group intermolecularly bonded solvent molecule (ESI Fig. S8 and S9†). This causes *enol* form more stable with respect to the *keto* form. The n → σ* interactions of salicylate anion moiety explicitly solvated with two and three ACN/water molecules have been presented in ESI Fig. S6–S9,† and have been tabulated in Table 5. From the above analysis, it can be concluded that both intra- and intermolecular interactions are stronger in ACN compared to water molecules, when the solvent was at carboxylate position. Placement of solvent molecule at phenolic position leads to stronger intramolecular interaction but weaker intermolecular interaction.

**Table 5:** Explicit solvation hydrogen bond strength^a^

Salicylate anion species	n → σ* interaction (kcal mol^−1^)		Salicylate anion species	n → σ* interaction (kcal mol^−1^)	
	Intra-molecular	Inter-molecular		Intra-molecular	Inter-molecular
1 ACN (c)	74.3	6.45c, 4.74c	1 H_2_O(c)	51.04	6.95c, 2.68c
1 ACN (p)	74.3	6.81p	1 H_2_O(p)	46.71	15.91p
2 ACN	46.66	3.84c, 4.23c	2 H_2_O	—	6.87c, 2.1c, 9.63p
3 ACN	51.27	6.46c, 7.11c, 2.38p	3 H_2_O	—	7.94c, 8.72p, 15.04c

a c = at carboxylic position, p = at phenolic position.

Further, in the excited state, the stabilization energy due to overall n → σ* interactions between salicylate anion and solvent molecule at the carboxylic position is found to be 1.78 kcal mol^−1^ in ACN and 6.52 kcal mol^−1^ in water. It suggests that this interaction leads to comparatively more charge transfer from salicylate anion to water carboxylate position, leading to an increase in the non-radiative transitions and hence acting as a probable cause for quenching in fluorescence intensity.

The overall NBO analysis reveals that the n → σ* intramolecular interaction is stronger in the *keto* form compared to the *enol* form in both solvents under the implicit solvation model. In the explicit solvation model, the n → σ* intramolecular interaction diminishes, while the n → σ* intermolecular interaction increases with the addition of solvent molecules. This shift results in a noticeable reduction in IMHB. Nevertheless, the IMHB remains stronger in ACN than in water across both solvation models.

### 3.5 IR spectra

To comprehend the quenching of salicylate anion fluorescence in water, computational IR spectra were also analyzed to interpret the effect of solvent polarity (implicit) and I_er_MHB (explicit) on IMHB. It provides a theoretical framework for predicting and inferring how the vibrational modes of the solvent affect the *enol–keto* structure of salicylate molecules in the ground and excited states. For computational reference, the IR spectra of a single molecule of ACN and water are shown in ESI Fig. S10.†

#### 3.5.1 Implicit solvation IR analysis

**Fig. 13:**
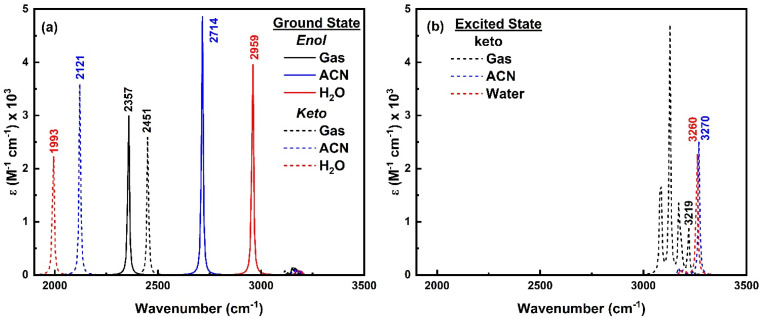
IR spectra of *enol* (solid line) and *keto* (dashed line) form of salicylate anion in (a) ground state and (b) excited state in the implicit environment.

The IR spectra of *enol* and *keto* forms of salicylate anion in the implicit environment in the ground state are shown in ESI Fig. S11,† while Fig. 13 displays the same IR spectra in the region 1900 cm^−1^ to 3500 cm^−1^ wavenumbers, focusing on IMHB while taking O–H stretching vibrations into consideration. Fig. 13a displays that, the O_d_-H stretch in *enol* form appears at 2357 cm^−1^ in the gaseous phase, however in implicit solvation, the O_d_-H vibration moves to 2714 cm^−1^ in ACN and 2959 cm^−1^ in water. This shift to higher wavenumbers suggests that the O_d_-H bond is becoming stronger in the polar solvent phase due to reduced IMHB (Table 1) because the solvent molecules are stabilizing the molecule through solvation and potentially forming intermolecular hydrogen bonds. In contrast, in the *keto* form of salicylate anion, the H-O_a_ stretch appears at 2451 cm^−1^ in the gaseous phase and shifts to 2121 cm^−1^ in ACN and 1993 cm^−1^ in water. The shift to lower wavenumbers indicates that the H-O_a_ bond is becoming weaker in the presence of solvent molecules. This weakening is likely due to the formation of potential strong intermolecular hydrogen bonds between the solvent molecules and the *keto* form. The solvent effectively pulls electron density away from the H-O_a_ bond, making it easier to stretch. It appears that in solvents, intensity of O–H stretching mode vibrations is higher compared to gas. As the intensity of a vibrational mode depends on the change in dipole moment, dielectric environment and hydrogen bonding interactions, ACN, with its moderate polarity and weaker hydrogen bonding compared to water, enhances the dipole change during vibrational transitions, leading to higher IR intensities for both forms. Water, with its strong hydrogen bonding capabilities, stabilizes the molecular forms and reduces the dipole fluctuations, resulting in lower IR intensities, especially for the *keto* form. Below 2000 cm^−1^ wavenumber shows a very meagre effect in the benzene ring and C–H vibrations (ESI Fig. S11†).

As only the *keto* form of salicylate molecule exists in the excited state (Fig. 3a), the implicit IR spectra in the excited state (Fig. 13b) show the H-O_a_ vibrations of the *keto* form occur at 3219 cm^−1^ in the gas phase, 3270 cm^−1^ in ACN, and 3260 cm^−1^ in water, showing a blue shift with increasing solvent polarity. Compared to the ground state, in the excited state, the O–H vibrations blue-shifted by Δ*ν̄* – 1267 cm^−1^ in water and 1149 cm^−1^ in ACN (*i.e.* appear at ∼3200 cm^−1^). The IR intensity of these vibrations increases with the solvent's polarity. Further, the C–H vibrations which are very intense in the gaseous phase, diminish in the solvent phase. This indicates that polar solvents stabilize the *keto* form, resulting in higher vibrational frequencies and enhanced IR intensities due to stronger solvent-induced dipole moment changes.

#### 3.5.2 Explicit solvation IR analysis

**Fig. 14:**
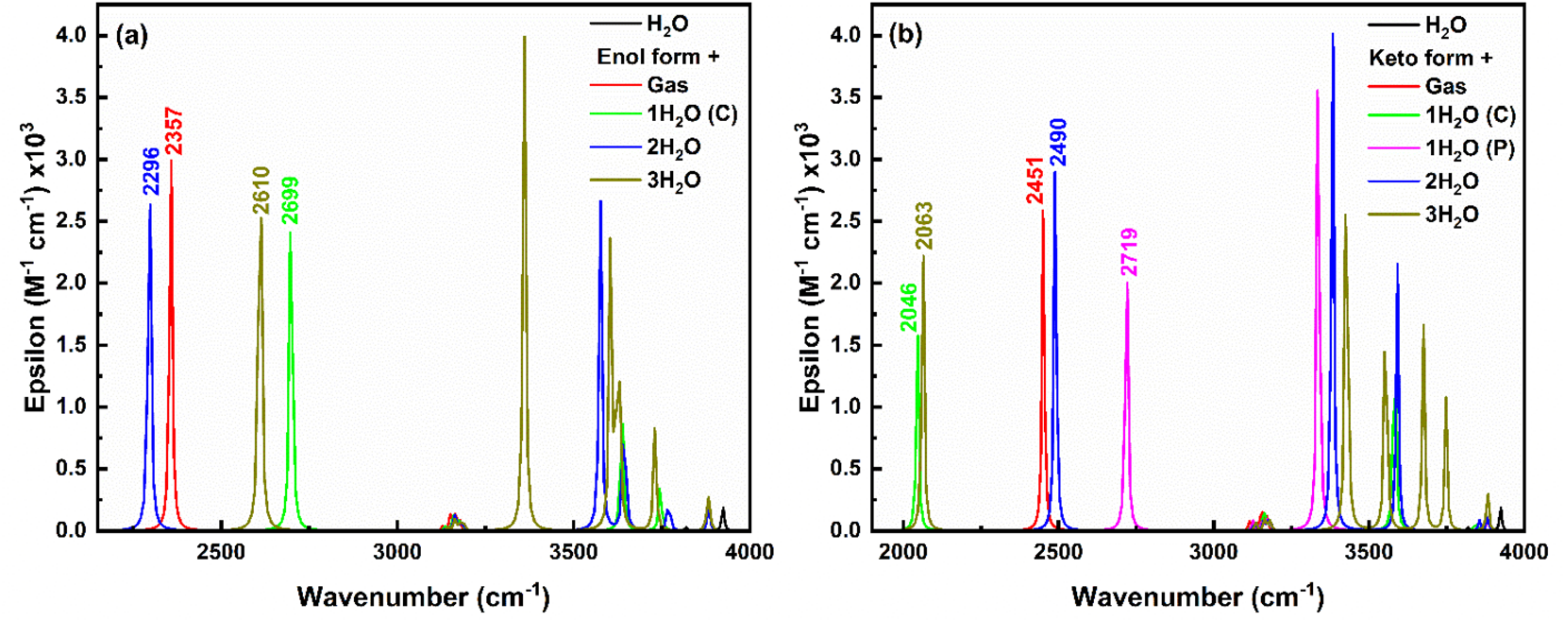
IR spectra of (a) *enol* form, and (b) *keto* form of salicylate anion explicitly solvated with water molecules.

Further, to understand the effect of explicit solvation dynamics on the vibrational spectra of *enol–keto* structures in the ground and excited states, IR spectra of salicylate anion with placement of solvent molecules (ACN/water) at various sites are shown in Fig. 14 and ESI Fig. S12,† respectively. Fig. 14a illustrates that the O_d_-H stretching vibrations of the *enol* form in the gas phase occur at 2357 cm^−1^. This vibration blue-shifts to 2699 cm^−1^ when single water is positioned at the carboxylate position, due to stabilization of the carboxylate's negative charge, leading to a stronger O_d_-H bond (less IMHB). Another water molecule at the phenolic position enhances the IMHB leading to a weaker O_d_-H bond and a red shift to a lower wavenumber. The formation of I_er_MHB with water molecules increases the O_d_-H bond length, resulting in a red shift to 2296 cm^−1^. Adding the third water molecule stabilizes the entire system, reducing the extent of IMHB involving the O_d_-H bond, and causing a blue shift to 2610 cm^−1^. It is worth noting that the IR intensity continuously decreases with the increasing number of solvent molecules.

In the *keto* form (Fig. 14b), H-O_a_ vibration occurs at 2451 cm^−1^ in gas phase, which red-shifts to 2046 cm^−1^ with single water at the carboxylate position due to strong I_er_MHB. Similarly, single water at phenolic position pulls electron density away from the H-O_a_ bond, leading to stronger H-O_a_ bond, resulting in a blue shift to 2719 cm^−1^. When water molecules are present at both the carboxylate and phenolic positions, the combined effect results in a slight red shift to 2490 cm^−1^ compared to the gas phase but less so than with just one water molecule at the carboxylate. Adding a third water molecule likely increases the overall solvation and hydrogen bonding network around the *keto* form, causing a significant to red-shift (2063 cm^−1^), similar to the single water molecule at the carboxylate position but less extreme. The IR intensity follows the order: (2 water) > gas > (3 water) > (1 water at phenolic) > (1 water at carboxylate), indicating a decrease in intensity with increasing stabilization of the dipole moment by the solvent. Further, in the excited state, only the *keto* structure is stabilised and the H-O_a_ stretching vibrations corresponding to this excited *keto* form occur about 3219 cm^−1^ in the gas phase (ESI Fig. S13†). A red-shift (3129 cm^−1^) in this H-O_a_ vibration appears, when a single water molecule placed near to at carboxylate position. Whereas a blue shift of 3377 cm^−1^ and 3297 cm^−1^ appears when two and three water molecules placed respectively near the salicylate molecule. Also, the O–H stretching water peak appears in the range of 3000 to 4000 cm^−1^, indicating an increase in vibrational crowding.

The O–H asymmetric stretching vibrations of water initially at 3924 cm^−1^ split into more wavenumbers and shift to lower wavenumbers with increasing water molecules near the *enol* form of salicylate anion, showing the highest intensity at 3637 cm^−1^ (one water at carboxylate), 3576 cm^−1^ (two waters), and 3360 cm^−1^ (three waters). Similar observations are found for *keto* form with the increase in splitting in peaks and an average red-shift in the highest intense peak. Thus, it can be concluded that the O–H asymmetric stretching vibrations of water exhibit splitting and shifts to lower wavenumbers as more water molecules interact with the salicylate anion. This indicates more complex and stronger hydrogen bonding interactions as the number of water molecules increases. Interestingly, on increasing the water molecules, some new vibrations appear above 3000 cm^−1^, corresponding to OH asymmetric stretching vibrations of water molecules (Fig. 14). The intensity of these vibrations is very high as compared to bare water molecule vibration [O–H stretching vibrations ∼3924 cm^−1^, 3819 cm^−1^ and bending vibrations ∼1600 cm^−1^ of low intensities (ESI Fig. S12†)]. On adding water molecules, some very low-frequency vibrations below 1000 cm^−1^ also appear as librational vibrations. A bare ACN molecule shows stretching vibrations about ∼2250 cm^−1^ (C≡N) and 3100 cm^−1^ (C–H), while bending and twisting vibrations occur about 1350–1470 cm^−1^ (C–H) (ESI Fig. S12†). Compared to water, placing the ACN molecule at different positions in the salicylate molecule, subtle change appears in the IR spectra of the salicylate molecule as shown in ESI Fig. S8.† These results indicate that in the presence of water, O–H vibrations intensity of salicylate anion decreases and the intensity of O–H vibrations of water increases. On correlating strong n → σ* I_er_MHB interaction, of salicylate anion with water molecules (31.7 kcal mol^−1^) as compared to ACN Molecule(15.95 kcal mol^−1^) and increased O–H vibrational crowding of water molecules in the presence of salicylate anion with deceased O_d_-H stretching vibrations of salicylate anion, indicates that non-radiative transitions increase and energy transfer takes place from salicylate anion to water molecule yielding in fluorescence quenching. Further, stronger n → σ* IMHB interaction of *keto* forms in both the solvents indicates equal probability of stabilization of *keto* form in water similar to in ACN, however, the polarity of the solvent stabilizes *keto* more in ACN as compared to water.

## 4. Conclusion

Implicit and explicit solvation approaches in simulated environments of ACN and water underscore their significant impact on the electronic properties of the salicylate anion. Implicit solvation results reveal that the *enol* form of the salicylate anion is more stable in the ground state, while only the *keto* form is stable in the excited state in both solvents. In both ACN and water, the absorption transitions are of π → π* character with nearly equal transition probabilities. However, the emission maximum is significantly red-shifted in water by 6 nm (∼416 cm^−1^) with a higher transition probability, leading to a large Stokes shift of approximately 10 000 cm^−1^ due to ESIPT. The slightly higher oscillator strength, dipole moment, and polarizability of the emission transition in water compared to ACN suggest that the solvent's polarity enhances the emission intensity with a red-shift. These findings indicate that solvent polarity alone cannot explain the stability of the *keto* form in the ground state in ACN or the fluorescence quenching observed in water.

In explicit solvation, placing solvent molecules near the phenolic and carboxylic functional groups of the salicylate anion revealed that I_er_MHB modulates the IMHB within the salicylate anion, impacting the stability of *enol–keto* tautomerization. I_er_MHB with water at the phenolic group stabilizes the *keto* form more effectively than with ACN molecules, suggesting a similar likelihood of *keto* form stabilization in both solvents. The stability of the salicylate anion increases with the addition of more solvent molecules, indicating the presence of various hydrogen-bonded salicylate anion oligomers, consistent with experimental findings. Notably, IMHB strength is greater in ACN, contributing to the enhanced stabilization of the *keto* form in ACN observed in absorption spectra. NBO analysis shows that n → σ* transitions are involved in both intermolecular and intramolecular processes. Furthermore, the I_er_MHB interaction energy at the carboxylic group of the salicylate anion is significantly higher in water compared to ACN.

Simulated IR spectra in implicit solvation showed a red shift in O_d_-H vibrations for the *enol* form and a blue shift in H-O_a_ vibrations for the *keto* form, indicating enhanced intra- and intermolecular hydrogen bonding, respectively. Additionally, in explicit solvation, there was an increase in the stretching vibrational frequencies of water molecules and a rise in very low vibrations, suggesting increasing phonon mode vibration. This results in excitation energy transfer from salicylate ions to water molecules non-radiatively *via* the n → σ* pathway through I_er_MHB. These explicit solvation findings align accurately with experimental results, predicting the stabilization of the hydrogen-bonded *keto* form in ACN, and explaining the fluorescence quenching observed in water.

## Data availability

The data that support the findings of this study are available from the corresponding author upon reasonable request.

## Conflicts of interest

There are no conflicts to declare.

## Supplementary Material

RA-014-D4RA04606D-s001
