# Supplementary material for: The effect of polarity and hydrogen bonding on the electronic and vibrational structure of the salicylate anion in acetonitrile and water: implicit and explicit solvation approaches
Source: RSC Adv. 2024 Sep 18;14(40):29569–87. doi: 10.1039/d4ra04606d (PMC11409446; doi:10.1039/d4ra04606d)
Supplement: RA-014-D4RA04606D-s001 [file RA-014-D4RA04606D-s001.pdf]

Supplementary Information (SI) for RSC Advances.
This journal is © The Royal Society of Chemistry 2024

ELECTRONIC SUPPLEMENTARY INFORMATION

The Effect of Polarity and Hydrogen Bonding on the Electronic and Vibrational Structure of Salicylate Anion in Acetonitrile and Water: Implicit and Explicit Solvation Approaches

Siddharth Mall Bishen[1,2], Meena Adhikari[2], Sandeep Pokharia[3], Hirdyesh Mishra[2]*

[1]Department of Physics, Institute of Science, Banaras Hindu University, Varanasi 221005, India

[2]Physics Section, MMV, Department of Physics, Banaras Hindu University, Varanasi 221005, India.

[3]Chemistry section, MMV, Banaras Hindu University, Varanasi 221005, India.

Table S1: GS optimized properties of salicylate anion at different functionals in implicit ACN

	B3LYP		CAM-B3LYP	M06-2X	PBE0
	Without GD3	With GD3			
**Energy (Hartree)**	-495.75660	-495.76679	-495.53625	-494.56030	-495.23136
**Dipole Moment (D)**	9.887	9.907	9.982	9.731	9.318

Table S2: GS optimized coordinates of *enol* form of salicylate anion in implicit ACN at B3LYP/6-311++G(d,p) without GD3 correction.

Tag	Symbol	NA	NB	NC	Bond	Angle	Dihedral	X	Y	Z
1	C	-	-	-	-	-	-	2.578373	-0.12692	0.000132
2	C	1	-	-	1.390262	-	-	1.82109	1.038992	-4E-06
3	C	2	1	-	1.403359	120.1752	-	0.419442	0.969722	-0.00013
4	C	3	2	1	1.416101	119.9476	0	-0.22606	-0.2907	-0.00013
5	C	4	3	2	1.400084	118.5055	0	0.564461	-1.44626	0.00001
6	C	5	4	3	1.391687	121.6421	0	1.954564	-1.37988	0.000139
7	H	1	2	3	1.085316	119.4335	-180	3.661582	-0.05932	0.000232
8	H	2	1	6	1.08488	120.9724	-179.999	2.297063	2.013884	-2.7E-05
9	C	4	3	2	1.519606	120.7449	179.9937	-1.74262	-0.38682	-0.00012
10	H	5	4	3	1.084098	117.7971	179.9978	0.058405	-2.405	0.00005
11	H	6	5	4	1.08439	120.4161	179.9986	2.547546	-2.28777	0.000266
12	O	9	4	3	1.244692	119.0121	179.9877	-2.27623	-1.51133	-0.00035
13	O	9	4	3	1.283713	115.5454	-0.04257	-2.36835	0.734066	0.000737
14	O	3	2	1	1.348126	119.1762	-179.998	-0.29507	2.112929	-0.00032
15	H	14	3	2	1.022229	103.2417	179.9952	-1.26297	1.784111	-0.00046
16	-	4	3	2	0.368254	10.75135	0.100983	0	0	0

Table S3: GS optimized coordinates of keto form of salicylate anion in implicit ACN at B3LYP/6-311++G(d,p) without GD3 correction

Tag	Symbol	NA	NB	NC	Bond	Angle	Dihedral	X	Y	Z
1	C	-	-	-	-	-	-	-2.57921	-0.0964	-7.3E-05
2	C	1	-	-	1.384274	-	-	-1.81698	1.059117	0.00005
3	C	2	1	-	1.424654	121.375	-	-0.39323	1.00851	-6.1E-05
4	C	3	2	1	1.437769	116.868	-0.00398	0.210586	-0.29632	-0.00022
5	C	4	3	2	1.403275	120.3511	0.000344	-0.59062	-1.44839	-0.00026
6	C	5	4	3	1.387259	121.4623	0.003745	-1.9755	-1.36722	-0.00024
7	H	1	2	3	1.086254	119.2735	-179.999	-3.66264	-0.01803	0.000003
8	H	2	1	6	1.085625	120.7068	-179.996	-2.29089	2.035844	0.00024
9	C	4	3	2	1.48559	119.794	-179.987	1.690609	-0.42481	-3.8E-05
10	H	5	4	3	1.084821	118.1133	-179.996	-0.09693	-2.41437	-0.00032
11	H	6	5	4	1.084026	120.7099	179.9989	-2.58268	-2.26524	-0.00029
12	O	9	4	3	1.227863	123.8276	179.9545	2.283375	-1.50011	0.000657
13	O	9	4	3	1.332215	114.8966	-0.01001	2.35386	0.730566	0.000053
14	O	3	2	1	1.301864	121.7701	179.9957	0.331104	2.090266	-2.9E-05
15	H	13	9	4	1.060749	105.7985	-0.00205	1.612444	1.489177	-6.7E-05
16	-	4	3	2	0.363531	10.56763	-0.15131	0	0	0

Table S4: GS optimized coordinates of *enol* form of salicylate anion in implicit H_2O at B3LYP/6-311++G(d,p) without GD3 correction

Tag	Symbol	NA	NB	NC	Bond	Angle	Dihedral	X	Y	Z
1	C	-	-	-	-	-	-	2.576449	-0.13529	-0.00011
2	C	1	-	-	1.389111	-	-	1.823773	1.032236	-0.0002
3	C	2	1	-	1.399622	120.0258	-	0.425802	0.964276	0.000102
4	C	3	2	1	1.41328	120.3457	-0.01764	-0.22815	-0.28861	0.000123
5	C	4	3	2	1.402154	118.2603	0.015827	0.559518	-1.44861	0.000177
6	C	5	4	3	1.38951	121.5604	-0.00472	1.947579	-1.38518	0.000112
7	H	1	2	3	1.084544	119.4271	-179.998	3.659104	-0.07131	-0.00031
8	H	2	1	6	1.084104	121.087	-179.994	2.30078	2.005758	-0.00047
9	C	4	3	2	1.508026	121.0681	-179.979	-1.73335	-0.38081	-0.00002
10	H	5	4	3	1.083228	118.3091	179.9942	0.059108	-2.40933	0.00029
11	H	6	5	4	1.083616	120.3015	179.9967	2.536461	-2.29481	0.000209
12	O	9	4	3	1.255629	119.3963	179.9859	-2.28163	-1.51041	-0.00027
13	O	9	4	3	1.283753	116.748	-0.01085	-2.38014	0.7281	0.000057
14	O	3	2	1	1.363853	118.4304	179.9964	-0.28099	2.1307	0.000079
15	H	14	3	2	1.006721	104.1001	179.9937	-1.24313	1.834458	0.000216
16	-	4	3	2	0.367891	10.76438	-0.08431	0	0	0

Table S5: GS optimized coordinates of *keto* form of salicylate anion in implicit H_2O at B3LYP/6-311++G(d,p) without GD3 correction

Tag	Symbol	NA	NB	NC	Bond	Angle	Dihedral	X	Y	Z
1	C	-	-	-	-	-	-	2.57719	-0.0873	0.00009
2	C	1	-	-	1.384264	-	-	1.809354	1.064485	-0.0001
3	C	2	1	-	1.41774	121.0466	-	0.393122	0.999117	-7.9E-05
4	C	3	2	1	1.432065	117.459	-0.00477	-0.20793	-0.30071	0.000044
5	C	4	3	2	1.404957	120.1965	0.004025	0.597662	-1.45176	0.000222
6	C	5	4	3	1.385168	121.1836	-0.00075	1.979779	-1.35987	0.000267
7	H	1	2	3	1.085391	119.246	-179.999	3.659319	-0.00321	0.00009
8	H	2	1	6	1.084784	120.7661	-179.999	2.277127	2.043231	-0.00026
9	C	4	3	2	1.476539	119.6842	-179.996	-1.67914	-0.426	-1.3E-05
10	H	5	4	3	1.084115	118.5532	179.9986	0.114617	-2.42231	0.000335
11	H	6	5	4	1.083421	120.6007	179.9997	2.591944	-2.25377	0.000409
12	O	9	4	3	1.235679	124.1801	-180	-2.28409	-1.50347	0.000085
13	O	9	4	3	1.334358	115.393	-0.00346	-2.35158	0.726534	-0.00011
14	O	3	2	1	1.318864	121.4646	-179.994	-0.34642	2.091122	-0.00038
15	H	13	9	4	1.07031	105.6174	-0.01086	-1.60644	1.494869	0.000076
16	-	4	3	2	0.365596	9.846425	-0.01348	0	0	0

Table S6: ES optimized coordinates of keto form of salicylate anion in implicit ACN

Tag	Symbol	NA	NB	NC	Bond	Angle	Dihedral	X	Y	Z
1	C	-	-	-	-	-	-	2.638501	-0.15374	0.000926
2	C	1	-	-	1.420987	-	-	1.87067	1.041933	0.000873
3	C	2	1	-	1.420231	121.4054	-	0.450806	1.009659	-0.00025
4	C	3	2	1	1.473691	119.851	0.051338	-0.25348	-0.28485	-0.00025
5	C	4	3	2	1.408648	116.9636	-0.09206	0.544131	-1.44592	-0.00112
6	C	1	2	3	1.391897	118.4105	0.007037	1.966205	-1.37251	-0.00031
7	H	1	6	5	1.083353	120.898	179.9913	3.721183	-0.11563	0.001671
8	H	2	1	6	1.085014	121.3581	-179.952	2.345192	2.017681	0.001033
9	C	4	3	2	1.457428	123.021	179.9364	-1.70647	-0.39849	-6E-06
10	H	5	4	3	1.082779	118.982	-179.946	0.060482	-2.41468	-0.00203
11	H	6	1	2	1.085419	119.7769	179.978	2.530766	-2.29955	-0.00066
12	O	9	4	3	1.244323	123.3807	-179.967	-2.308	-1.48776	0.000595
13	O	9	4	3	1.372067	118.3272	-0.02853	-2.44972	0.754836	0.000831
14	O	3	2	1	1.286412	120.7776	-179.95	-0.23241	2.099648	-0.00125
15	H	13	9	4	0.990831	106.1319	0.106812	-1.79878	1.501845	-0.00064
16	-	4	3	2	0.381301	13.11699	0.112361	0	0	0

Table S7: ES optimized coordinates of keto form of salicylate anion in implicit H_2O at B3LYP/6-311++G(d,p) without GD3 correction

Tag	Symbol	NA	NB	NC	Bond	Angle	Dihedral	X	Y	Z
1	C	-	-	-	-	-	-	2.638868	-0.15463	-0.00069
2	C	1	-	-	1.413228	-	-	1.87397	1.033705	-0.00046
3	C	2	1	-	1.420006	121.7469	-	0.45419	1.008394	0.000178
4	C	3	2	1	1.478363	119.6492	-0.0183	-0.25412	-0.28924	0.000199
5	C	4	3	2	1.402934	116.7185	0.055448	0.543608	-1.4433	0.000785
6	C	1	2	3	1.392888	118.3062	-0.01402	1.965165	-1.37376	0.000015
7	H	1	6	5	1.082556	120.9622	179.998	3.72074	-0.11616	-0.00124
8	H	2	1	6	1.084652	121.1763	179.9624	2.350394	2.008123	-0.00038
9	C	4	3	2	1.444714	122.9072	-179.951	-1.69481	-0.39704	-0.00022
10	H	5	4	3	1.082722	119.2838	179.9515	0.067913	-2.41593	0.001622
11	H	6	1	2	1.08442	119.9432	-179.992	2.52581	-2.302	-1.9E-05
12	O	9	4	3	1.260753	124.157	-179.989	-2.32285	-1.49023	-4E-06
13	O	9	4	3	1.385657	118.9348	0.010512	-2.45383	0.762238	-0.00088
14	O	3	2	1	1.289274	120.7993	179.9625	-0.2256	2.103893	0.001109
15	H	13	9	4	0.991137	106.1154	-0.01613	-1.8079	1.513986	-0.00068
16	-	4	3	2	0.385016	12.67415	-0.10229	0	0	0

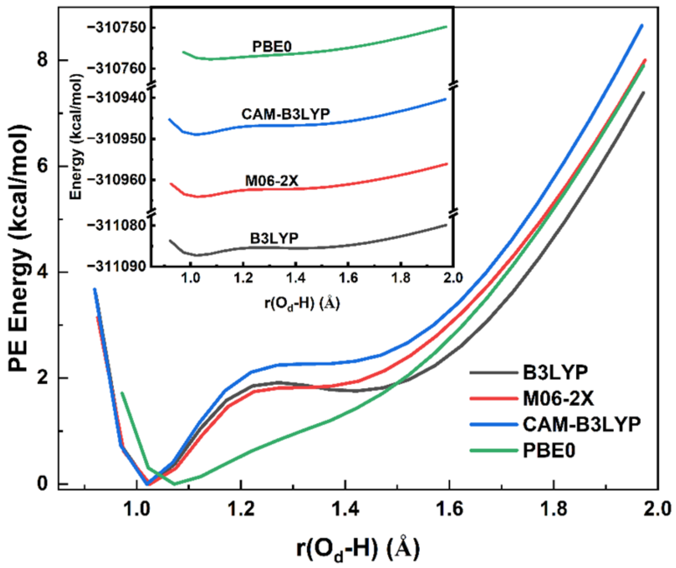

Fig. S1: GSIPT curve of Salicylate anion in implicit ACN computed at different basis sets.

Table S8: Comparison between different basis sets for Salicylate anion in implicit ACN

Transition	Species	B3LYP	CAM-B3LYP	M06-2X	PBE	Experimental
Absorption (nm) (f)	A	275.35 (0.1167)	260.3 (0.1273)	257.14 (0.1299)	306.47 (0.1071)	296
	A_T	318.89 (0.1503)	293.37 (0.1695)	287.07 (0.1697)	-	~330
Emission (nm) (f)	A_T	379.07 (0.2345)	354.53 (0.2853)	354.48 (0.2907)	425.52 (0.1858)	392

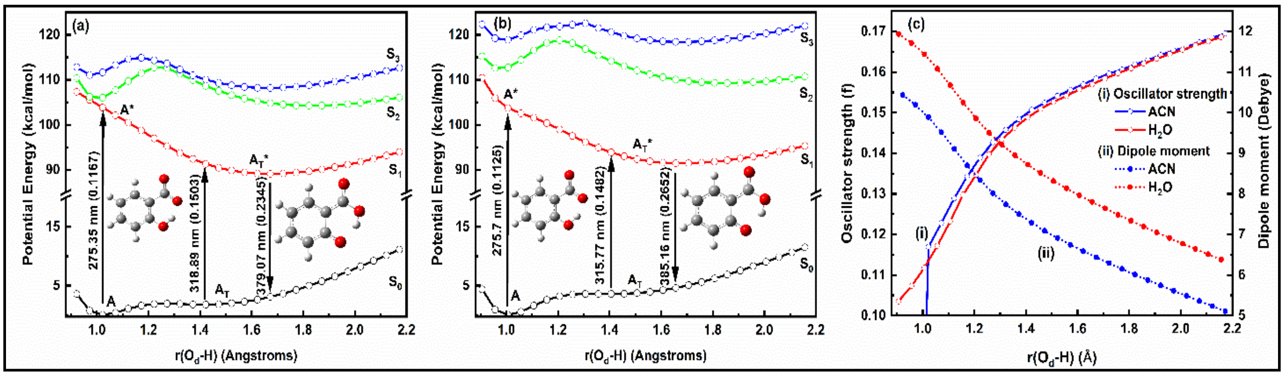

Fig. S2: PE curves of Salicylate anion in (a) ACN and (b) water; (c) Variation in (i) oscillator strength and (ii) transient dipole moment of salicylate anion in ACN and water with proton transfer for first singlet transition.

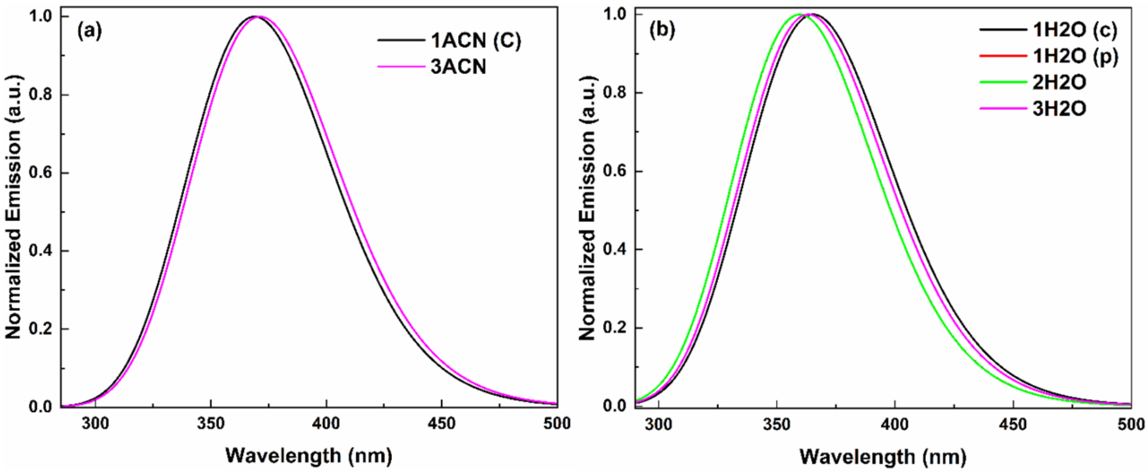

Fig. S3: Normalized emission of salicylate anion explicitly solvated with (a) ACN (b) water molecules.

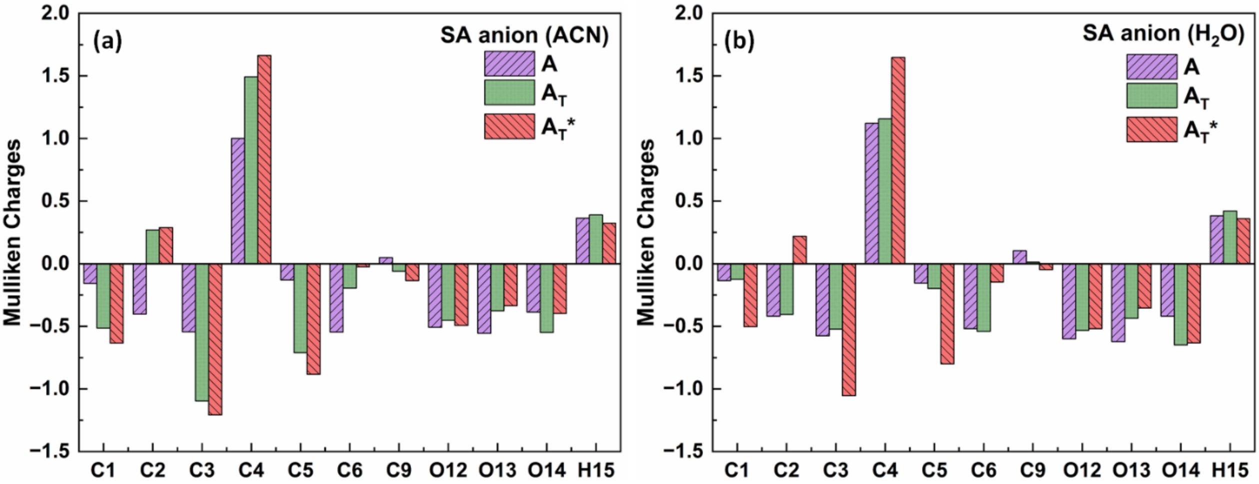

Fig. S4: Mulliken charge analysis of salicylate anion in implicit (a) ACN (b) water environment.

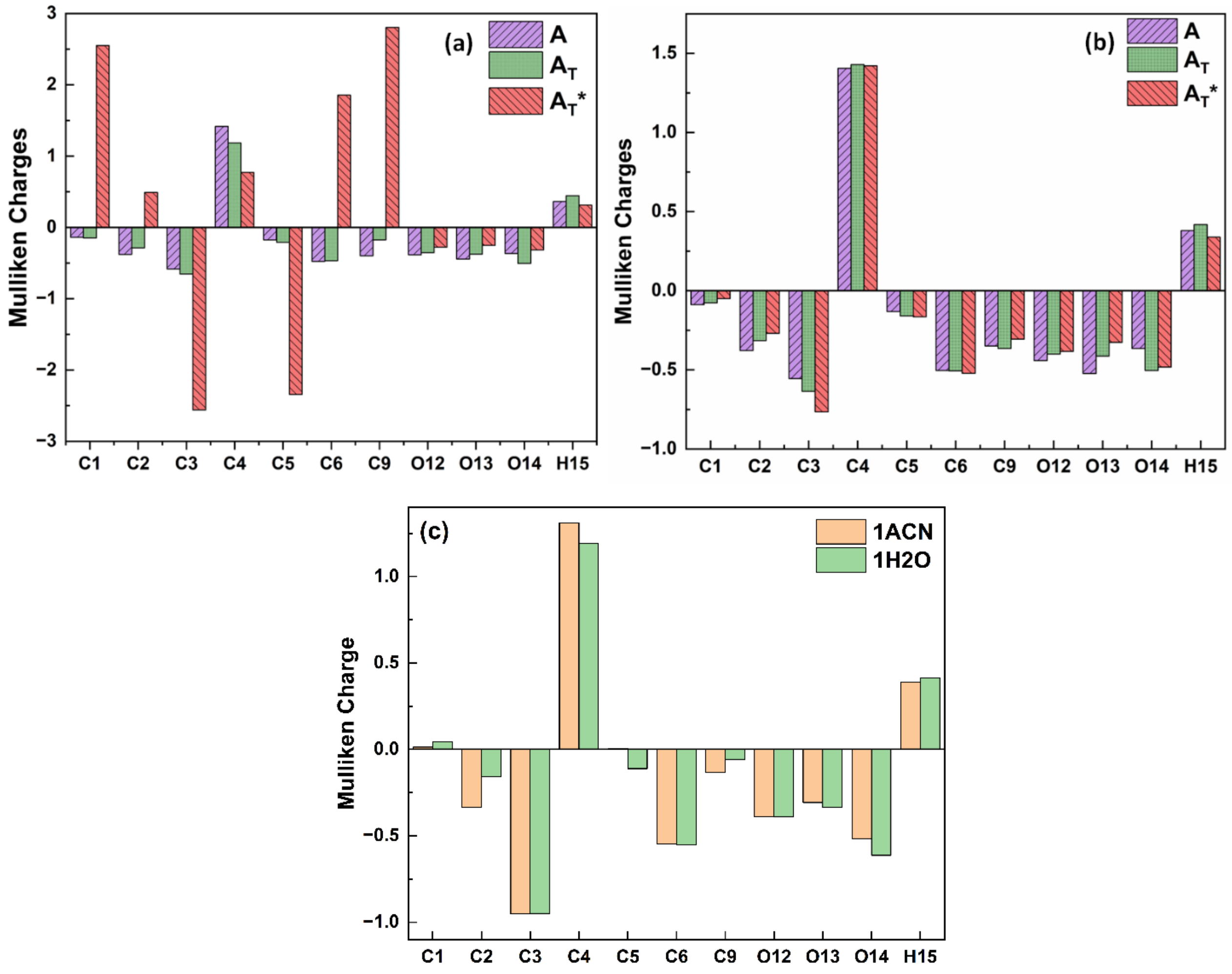

Fig. S5: Mulliken charge analysis of salicylate anion explicitly solvated with single (a) ACN (b) water molecule placed near the carboxylate position, (c) solvent molecule placed near the phenolic position.

Table S9: Hyperconjugative stabilization energies (($\Delta E_{ij}^{(2)}$) for selected NBO pairs from second-order perturbation theory analysis of the Fock matrix in the NBO analysis for the salicylate anion moiety.

SA anion Species	Donor NBO (i) (LP)	Acceptor NBO (j) (σ*)	$\Delta E_{ij}^{(2)}$ (kcal/mol)	
			ACN	Water
Enol	LP1(O12)	σ*(C4-C9)	2.02	1.98
		σ*(C9-O13)	1.99	1.57
	LP2(O12)	σ*(C3-C4)	0.56	0.51
		σ*(C4-C9)	16.81	15.06
		σ*(C4-O13)	21.72	20.88
	LP1(O13)	σ*(C4-C9)	4.80	4.44
		σ*(O14-H15)	5.32	4.31
	LP2(O13)	σ*(C4-C9)	6.92	7.32
		σ*(C9-O12)	16.53	17.46
		σ*(O14-H15)	45.79	34.30
	LP1(O14)	σ*(C3-C4)	6.33	6.04
Keto	LP1(O12)	σ*(C4-C9)	2.38	2.32
		σ*(C9-O13)	1.16	1.02
	LP2(O12)	σ*(C4-C9)	15.88	14.72
		σ*(C9-O13)	27.25	26.06
	LP1(O13)	σ*(C4-C9)	5.32	5.23
		σ*(C9-O12)	1.23	1.29
	LP(1)O14	σ*(C3-C4)	5.99	5.81
		σ*(O13-H15)	5.73	5.70
	LP2(O14)	σ*(C2-C3)	11.36	10.01
		σ*(C3-C4)	5.56	4.68
		σ*(O13-H15)	69.94	76.02

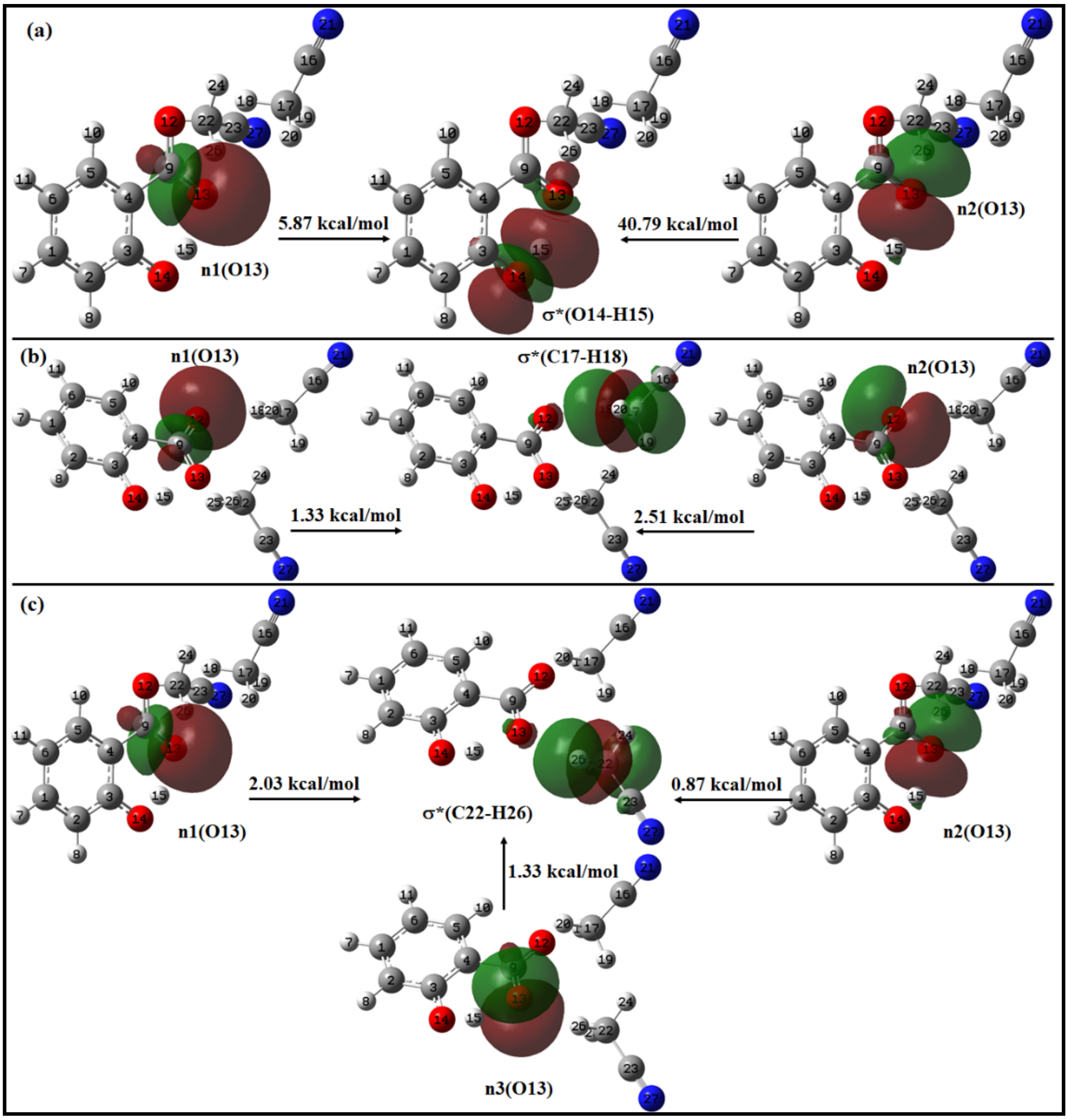

Fig. S6: Delocalization of charge from the lone pair orbital (n) to σ* molecular orbital: (a) within SA anion's enol form; between SA anion and (b) first ACN molecule (c) second ACN molecule, both situated near carboxylate group

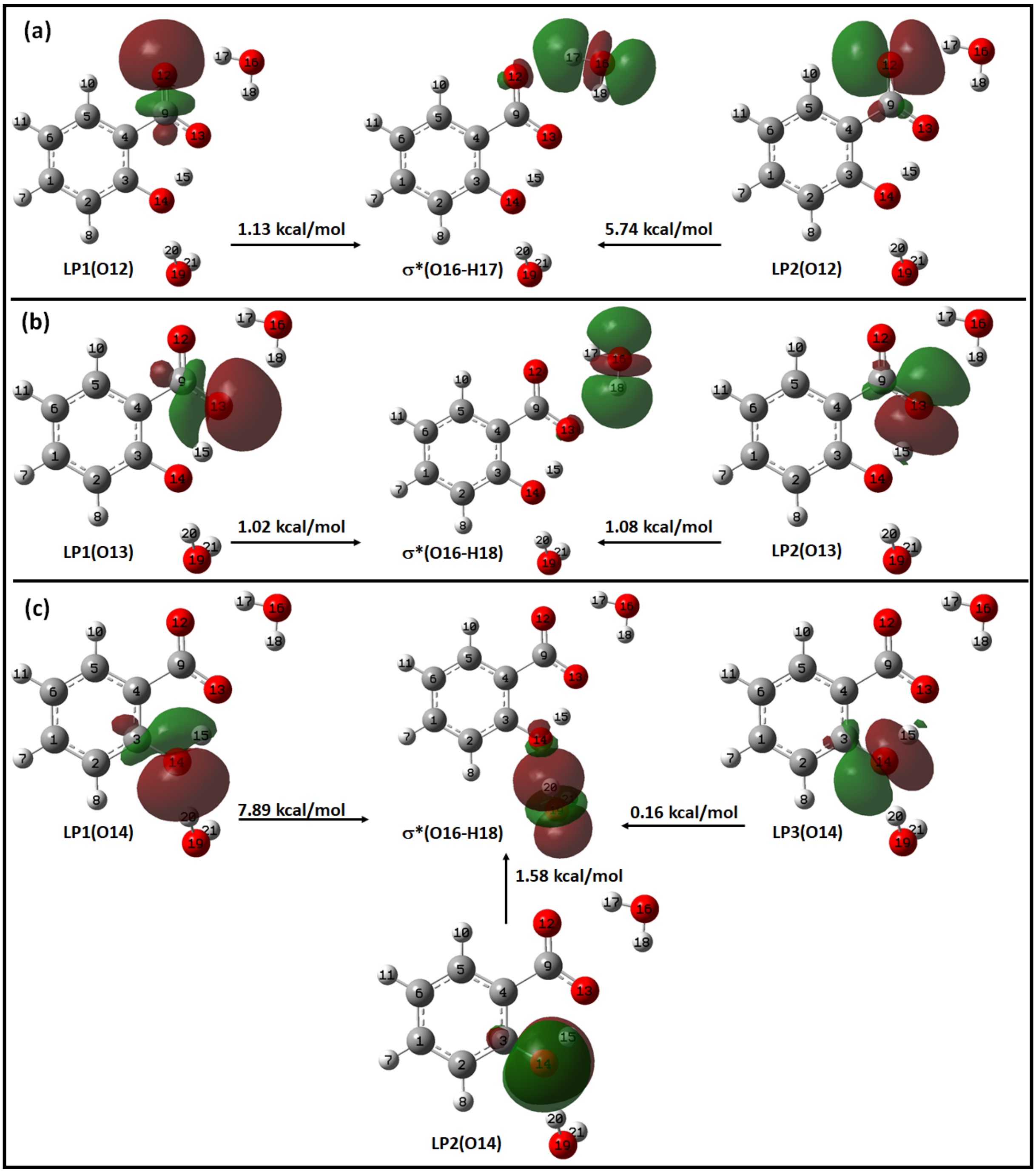

Fig. S7: Charge delocalization from the lone pair orbital (n) of oxygen atoms of SA anion's enol form to σ* molecular orbital of water molecule: (a) n(O12)→σ(O16-H17) (b) n(O13)→σ(O16-H18), and (c) n(O13)→σ(O16-H18) interactions.

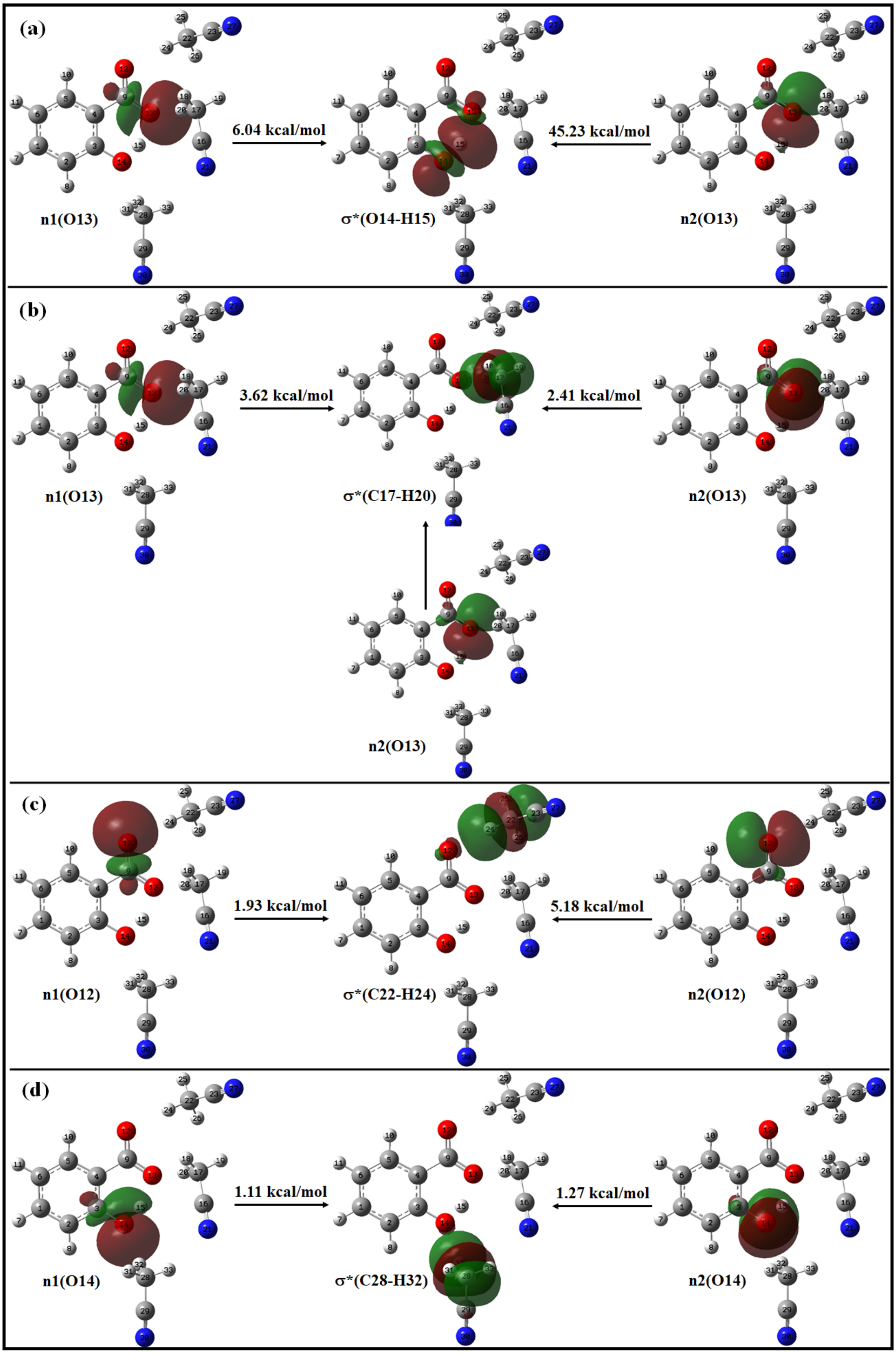
(a)
6.04 kcal/mol
45.23 kcal/mol
n1(O13)
σ*(O14-H15)
n2(O13)
(b)
3.62 kcal/mol
2.41 kcal/mol
n1(O13)
σ*(C17-H20)
n2(O13)
n2(O13)
(c)
1.93 kcal/mol
5.18 kcal/mol
n1(O12)
σ*(C22-H24)
n2(O12)
(d)
1.11 kcal/mol
1.27 kcal/mol
n1(O14)
σ*(C28-H32)
n2(O14)

Fig. S8: Delocalization of charge from the lone pair orbital (n) to σ* molecular orbital: (a) within SA anion's enol form; between SA anion and (b) first ACN molecule (b) second ACN molecule, both situated near carboxylate group, and (d) third ACN molecule near the phenolic position.

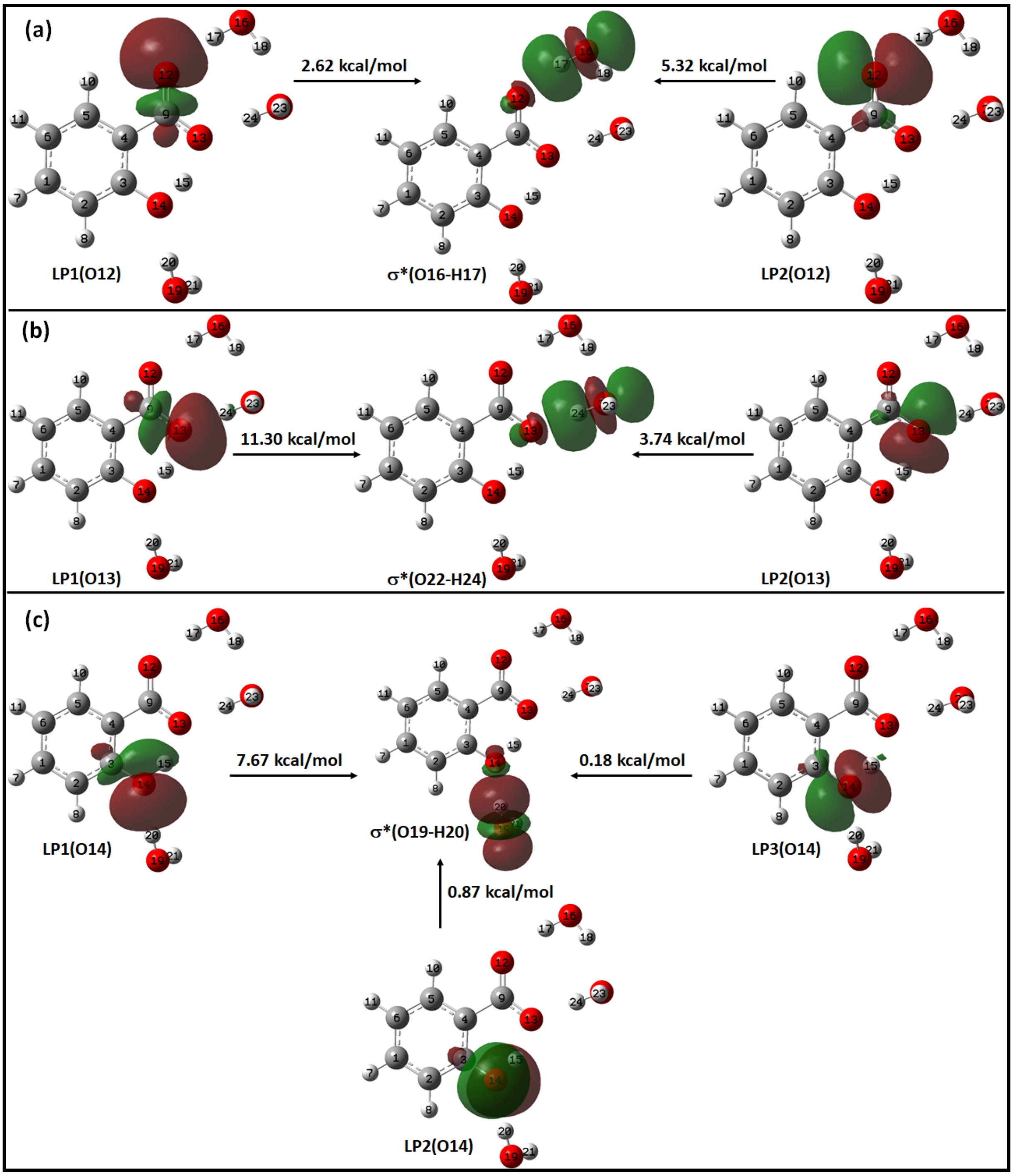

Fig. S9: Delocalization of charge from the lone pair orbital (n) of oxygen atoms of SA anion's enol form to σ* molecular orbital of: (a) water molecule near O12 atom (b) water molecule near O13 atom, at carboxylate positions, and (d) third water molecule near the phenolic oxygen O14 atom.

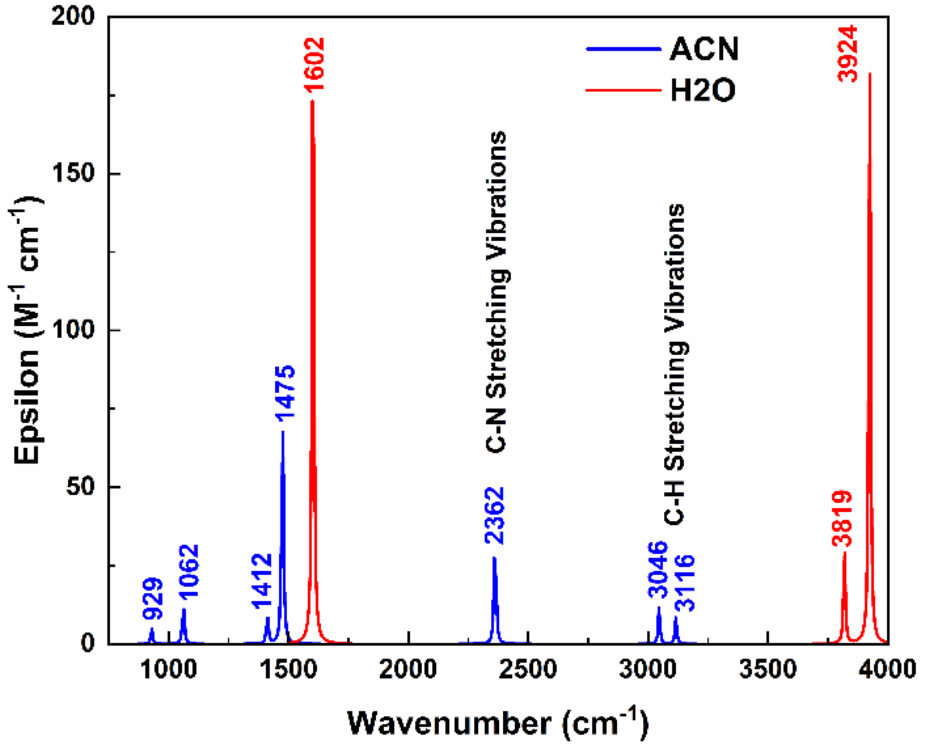

Fig. S10: Computational IR spectra of a single molecule of ACN and water in the ground state.

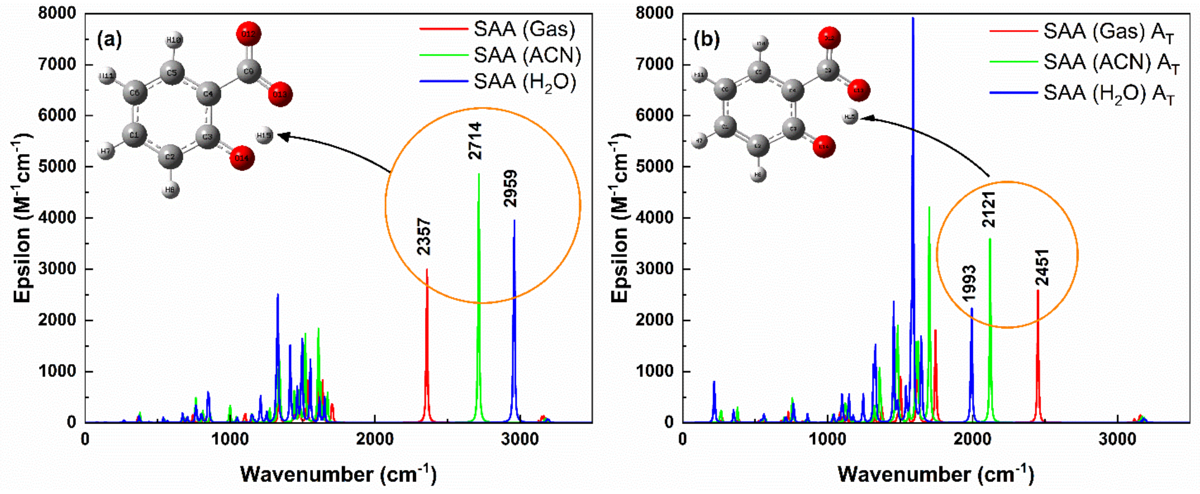

Fig. S11: Computational IR spectra of (a) *enol* form and (b) *keto* form of SA anion in the ground state in the implicit environment.

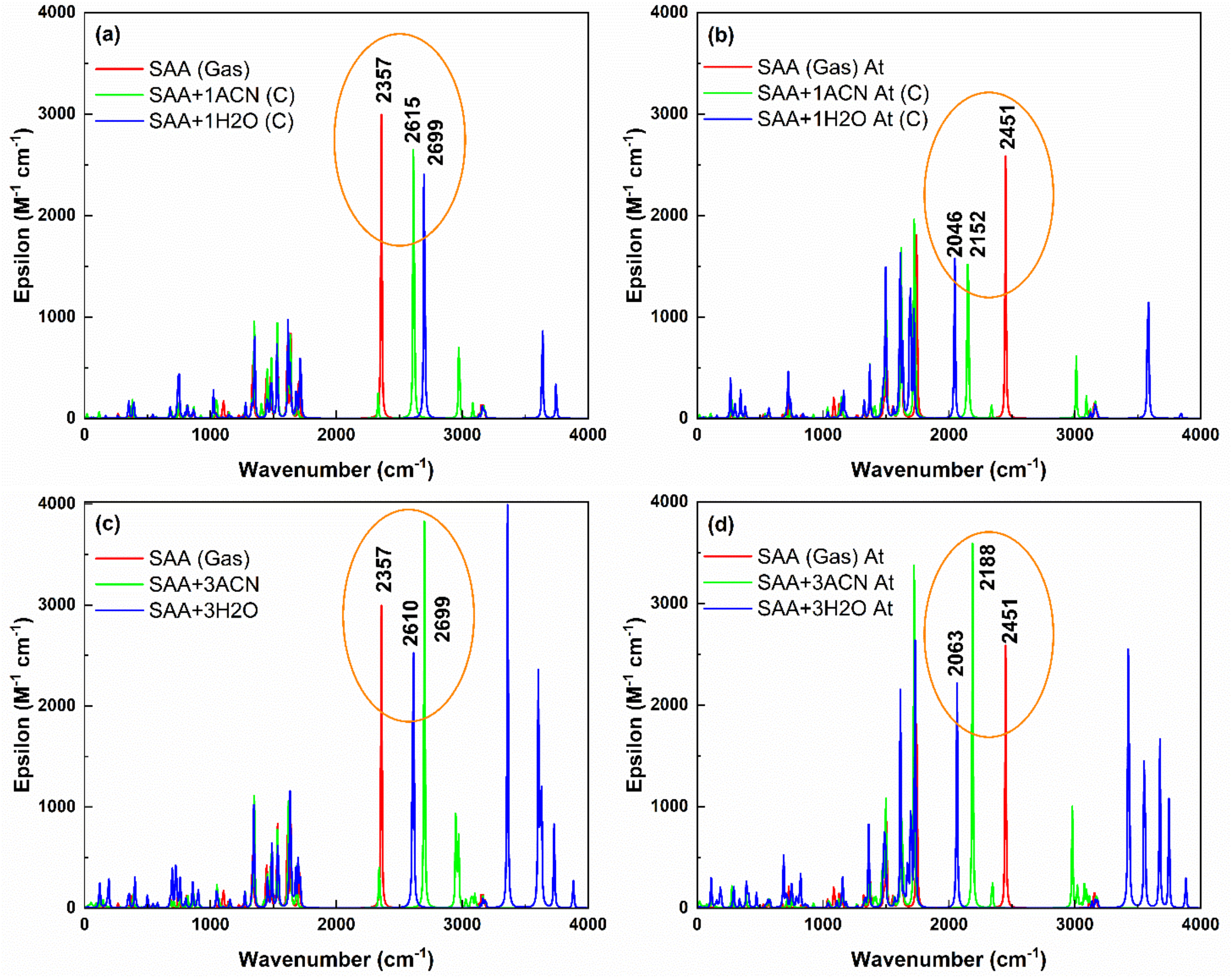

Fig. S12: Computational IR spectra of (a) enol form and (b) keto form of SA anion in ground state with one solvent molecule at carboxylate position; (c) enol form and (d) keto form with three solvent molecules.

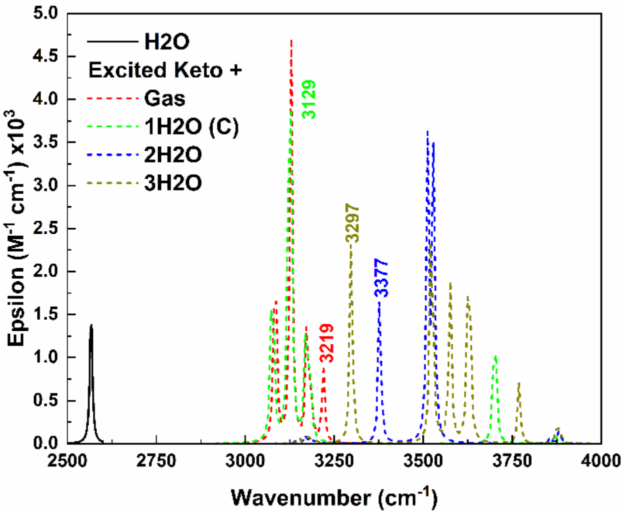

Fig. S13: Computational IR spectra of SA anion in excited state with increasing number of water molecule at various positions Numbered peaks correspond to H-O_a stretching vibrations.
